# Patterns of Oncogene Coexpression at Single-Cell Resolution Influence Survival in Lymphoma

**DOI:** 10.1158/2159-8290.CD-22-0998

**Published:** 2023-04-18

**Authors:** Michal Marek Hoppe, Patrick Jaynes, Fan Shuangyi, Yanfen Peng, Shruti Sridhar, Phuong Mai Hoang, Clementine Xin Liu, Sanjay De Mel, Limei Poon, Esther Hian Li Chan, Joanne Lee, Choon Kiat Ong, Tiffany Tang, Soon Thye Lim, Chandramouli Nagarajan, Nicholas F. Grigoropoulos, Soo-Yong Tan, Susan Swee-Shan Hue, Sheng-Tsung Chang, Shih-Sung Chuang, Shaoying Li, Joseph D. Khoury, Hyungwon Choi, Carl Harris, Alessia Bottos, Laura J. Gay, Hendrik F.P. Runge, Ilias Moutsopoulos, Irina Mohorianu, Daniel J. Hodson, Pedro Farinha, Anja Mottok, David W. Scott, Jason J. Pitt, Jinmiao Chen, Gayatri Kumar, Kasthuri Kannan, Wee Joo Chng, Yen Lin Chee, Siok-Bian Ng, Claudio Tripodo, Anand D. Jeyasekharan

**Affiliations:** 1 Cancer Science Institute of Singapore, National University of Singapore, Singapore, Singapore.; 2 Department of Pathology, Yong Loo Lin School of Medicine, National University of Singapore, Singapore, Singapore.; 3 Department of Haematology-Oncology, National University Health System, Singapore, Singapore.; 4 NUS Centre for Cancer Research, Yong Loo Lin School of Medicine, National University of Singapore, Singapore, Singapore.; 5 Division of Cellular and Molecular Research, National Cancer Centre Singapore, Singapore, Singapore.; 6 Division of Medical Oncology, National Cancer Centre Singapore, Singapore, Singapore.; 7 Department of Haematology, Singapore General Hospital, Singapore, Singapore.; 8 Department of Pathology, Chi-Mei Medical Center, Tainan City, Taiwan.; 9 Department of Hematopathology, Division of Pathology and Laboratory Medicine, The University of Texas MD Anderson Cancer Center, Houston, Texas.; 10 Department of Pathology and Microbiology, University of Nebraska Medical Center, Omaha, Nebraska.; 11 Department of Medicine, Yong Loo Lin School of Medicine, National University of Singapore, Singapore, Singapore.; 12 F. Hoffmann-La Roche Ltd, Basel, Switzerland.; 13 Wellcome MRC Cambridge Stem Cell Institute, Cambridge, United Kingdom.; 14 BC Cancer Research Centre, Vancouver, Canada.; 15 Genome Institute of Singapore, Agency for Science, Technology and Research, Singapore, Singapore.; 16 Singapore Immunology Network, Agency for Science, Technology and Research, Singapore, Singapore.; 17 Translational Molecular Pathology, The University of Texas MD Anderson Cancer Center, Houston, Texas.; 18 Tumor Immunology Unit, University of Palermo, Palermo, Italy.; 19 IFOM ETS – The AIRC Institute of Molecular Oncology, Milan, Italy.

## Abstract

Cancers often overexpress multiple clinically relevant oncogenes, but it is not known if combinations of oncogenes in cellular subpopulations within a cancer influence clinical outcomes. Using quantitative multispectral imaging of the prognostically relevant oncogenes *MYC*, *BCL2*, and *BCL6* in diffuse large B-cell lymphoma (DLBCL), we show that the percentage of cells with a unique combination *MYC*+*BCL2*+*BCL6*− (M+2+6−) consistently predicts survival across four independent cohorts (*n* = 449), an effect not observed with other combinations including M+2+6+. We show that the M+2+6− percentage can be mathematically derived from quantitative measurements of the individual oncogenes and correlates with survival in IHC (*n* = 316) and gene expression (*n* = 2,521) datasets. Comparative bulk/single-cell transcriptomic analyses of DLBCL samples and *MYC*/*BCL2*/*BCL6*-transformed primary B cells identify molecular features, including cyclin D2 and PI3K/AKT as candidate regulators of M+2+6− unfavorable biology. Similar analyses evaluating oncogenic combinations at single-cell resolution in other cancers may facilitate an understanding of cancer evolution and therapy resistance.

**Significance:**

Using single-cell–resolved multiplexed imaging, we show that selected subpopulations of cells expressing specific combinations of oncogenes influence clinical outcomes in lymphoma. We describe a probabilistic metric for the estimation of cellular oncogenic coexpression from IHC or bulk transcriptomes, with possible implications for prognostication and therapeutic target discovery in cancer.

*This article is highlighted in the In This Issue feature, p. 1027*

## Teaser

Quantitative multiplexed microscopy reveals that a subpopulation of cells that coexpress MYC and BCL2 without BCL6 govern clinical outcomes in diffuse large B-cell lymphoma, underscoring the importance of analyzing protein coexpression patterns at single-cell resolution.

## INTRODUCTION

Oncogene overexpression is common in cancer. The concomitant increase in oncogenic proteins (oncoproteins) influences both prognosis and treatment (1). Notable examples routinely assessed in clinical practice include HER2 in breast cancer and ALK in lung cancer. However, cancers often overexpress more than one oncogene. Whether multiple oncogenes interact at the single-cell level to influence clinical outcome remains an important unresolved question. This is particularly relevant because cancers are a heterogeneous mosaic of tumor cell subpopulations (2), and oncogenes show clinically significant intratumor heterogeneity (ITH) in expression (1). Clinical techniques for estimating oncogene overexpression in cancer (such as IHC) study them in isolation, and do not provide information on coexpression in subsets of cells within a tumor. It is therefore still not known if subsets of cells within a given cancer expressing specific combinations of oncogenes drive clinical phenotypes.

We aimed to address this question using multiplexed fluorescent IHC (mfIHC), a technique that can simultaneously and quantitatively evaluate a set of proteins with single-cell resolution. This allows measurement of single-cell oncogene coexpression from sufficient samples for robust multivariate correlations with clinical outcomes. We chose diffuse large B-cell lymphoma (DLBCL) as a model to evaluate the clinical impact of ITH in oncogene coexpression. DLBCL is the most common aggressive lymphoma worldwide (3), and overexpression of the oncogenes *MYC*, *BCL2*, and *BCL6* (4–6) influences pathogenesis and prognosis (7–9). However, there is significant variability among studies regarding the prognostic significance of these oncogenes, with debate on appropriate cutoff thresholds to define “positivity.” These considerations offer an ideal scenario to evaluate whether these oncogenes show differential coexpression at the single-cell level in DLBCL, and to investigate how they cooperate or influence each other at the cellular level to affect survival.

## RESULTS

### Physiologic Patterns of MYC, BCL2, and BCL6 Coexpression Are Disrupted in DLBCL

**Figure 1.**
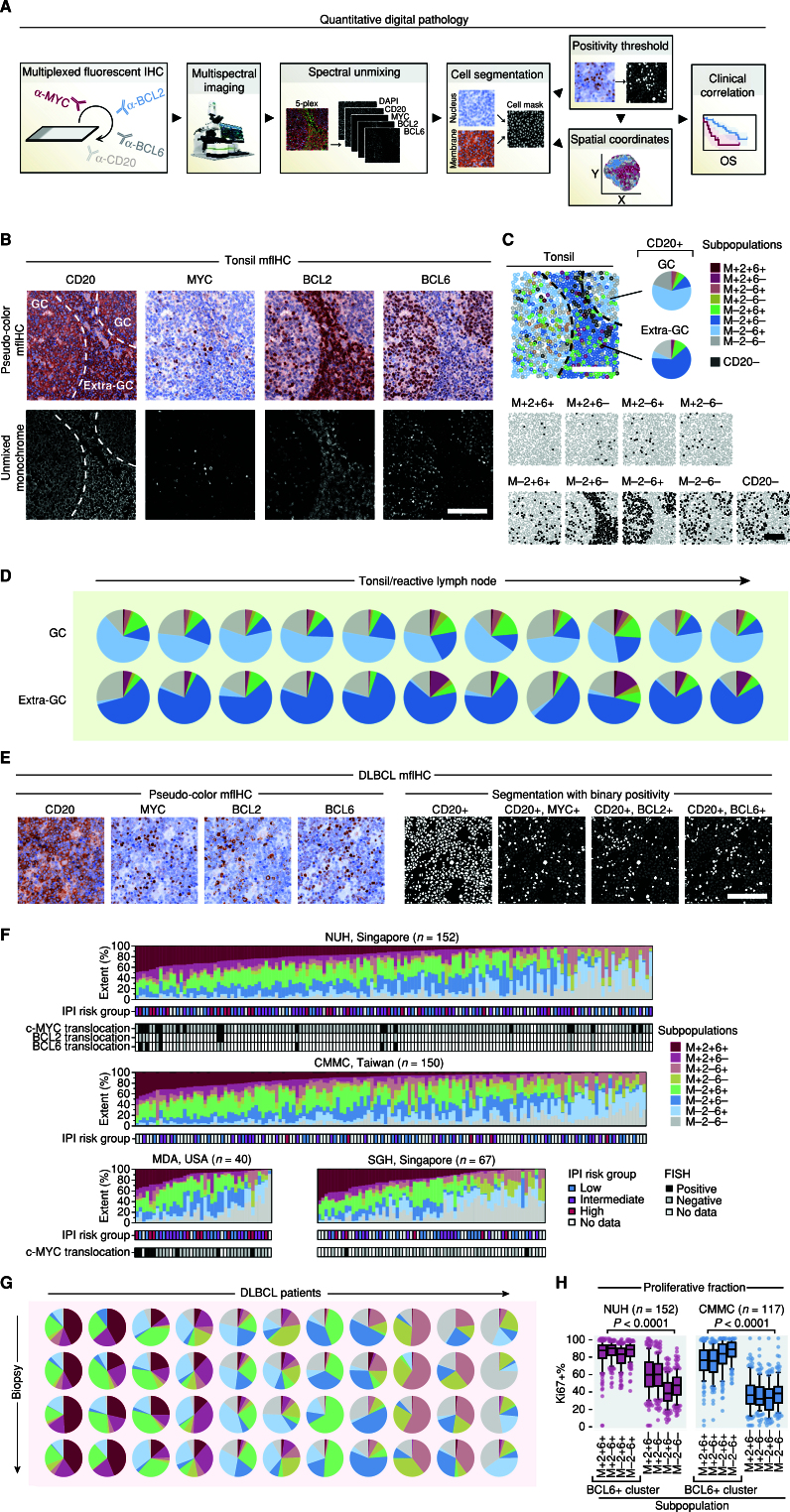
Quantitative single-cell analysis of MYC, BCL2, and BCL6 protein expression in B cells in nonmalignant tissues and diffuse large B-cell lymphoma. **A,** Schematic workflow of a quantitative digital pathology experiment. **B,** Spectrally unmixed multiplexed fluorescent images for CD20, MYC, BCL2, and BCL6 and nuclear counterstaining in tonsil tissue. The germinal center (GC) and extragerminal center (extra-GC) zones are indicated. **C,** Spatial map of MYC/BCL2/BCL6 subpopulations, i.e., possible permutations of MYC/BCL2/BCL6-positivity and -negativity within the CD20-positive cell population in a tonsil image. **D,** Quantitation of subpopulation extent within CD20-positive cells in tonsils and reactive lymph nodes resolved spatially between the GC and extra-GC zones. **E,** Example of pseudocolored MYC/BCL2/BCL6/CD20 mfIHC staining in diffuse large B-cell lymphoma (DLBCL; left). Cell segmentation and single oncogene positivity masks are shown within the CD20-positive cell population (right). **F,** Summary of percentage extent of subpopulations across patients from National University Hospital (NUH), Chi-Mei Medical Center (CMMC), MD Anderson (MDA), and Singapore General Hospital (SGH). Relevant clinicopathologic features are indicated; see also Supplementary Fig. S3. Patients were ordered arbitrarily according to extent of the triple-positive M+2+6+ subpopulation extent. IPI Risk Group, International Prognostic Index Risk Group; FISH, fluorescence *in situ* hybridization. **G,** Intrapatient spatial stability of subpopulations – proportion of subpopulations measured across four spatially distinct biopsies from the same patient (rows). Biopsy comparison overview is shown across 11 representative example DLBCL patients (columns). See also Supplementary Fig. S4A and S4B for a correlation analysis for all patients with multiple biopsies available. **H,** Proliferation analysis (i.e., Ki-67-positivity) among subpopulations in DLBCL samples. Proliferative BCL6-positive subpopulations are grouped. Median with interquartile range, whiskers denote 10th and 90th percentile. Mann–Whitney test (BCL6-positive vs. -negative subpopulations). All scale bars, 100 μm.

We first compared the coexpression of the oncogenes *MYC*, *BCL2*, and *BCL6* between reactive lymphoid tissue and DLBCL by mfIHC (Fig. 1A). Consistent with known physiology, BCL6 and BCL2 expression was restricted to reactive lymphoid germinal centers (GC) and extra-GC regions, respectively, whereas MYC showed sparse positivity in the GC CD20^+^ cells (ref. 10; Fig. 1B; Supplementary Fig. S1A; Supplementary Table S1). A binary ± map for each oncogene facilitates the quantitation of subpopulations of cells based on MYC BCL2 and BCL6 coexpression (Supplementary Fig. S1B). The repertoire of subpopulations defined by MYC/BCL2/BCL6 permutations in reactive lymphoid B cells was limited, with the single-positive M-2–6+ subpopulation being dominant within the GC and predominantly driving proliferative capacity (Supplementary Fig. S1C), and M-2+6− being dominant outside the GC (Fig. 1C). Hardly any cells displayed coexpression of all three oncogenes MYC, BCL2, and BCL6 in either compartment, consistent across several reactive lymphoid tissues analyzed (Fig. 1D; Supplementary Table S1).

In contrast, DLBCL cells frequently coexpress these three oncogenes (Fig. 1E and F; Supplementary Fig. S2; Supplementary Table S2). However, even within cases characterized by high overall levels of MYC, BCL2, and BCL6 expression, these three oncogenes were not always found in the same cells, underscoring ITH in DLBCL. The percentage of each subpopulation was variable between patients but was remarkably similar in overall distribution and clustering among four cohorts (Fig. 1F; Supplementary Fig. S3). The percentages of subpopulations were also stable across different tumor cores from the same patient, indicative of patient-specific subpopulation profiles (Fig. 1G; Supplementary Fig. S4A and S4B). These subpopulations were not consistently associated with clinicopathologic features such as age, gender, and International Prognostic Index (IPI) Risk Group, nor were they associated with MYC/BCL2/BCL6 translocation status (Supplementary Table S3; Fig. 1F), confirming previous observations that translocations do not account for the majority of MYC, BCL2, and BCL6 overexpression in DLBCL (11). Only subpopulations with BCL6 expression (irrespective of the coexpression of other oncogenes) showed Ki-67 expression in two DLBCL cohorts (Fig. 1H). This association was also observed in the context of reactive tonsil tissue, consistent with the role of BCL6 in B-cell proliferation (Supplementary Fig. S1C).

### Spatial Interaction of Oncogenic B-cell Subpopulations Is Clustered and Nonrandom

Single-cell–resolved image data with spatial coordinates enable the assessment of spatial interaction patterns of the eight MYC, BCL2, and BCL6 subpopulations. Analyzing spatial subpopulation data of the Singapore General Hospital (SGH) and MD Anderson (MDA) cohorts, we first applied a pair correlation function (PCF; refs. 12, 13), which quantifies how a point (cell) of interest is surrounded by other cells and can investigate whether each subpopulation tends to cluster or show a random (Poisson) distribution (Supplementary Fig. S5A). In immediate neighborhoods—defined here as a range from 0- to 250-μm radius of a given cell—the PCF graphs demonstrate that for both cohorts each subpopulation deviates from Poisson spatial patterning (PCF = 1; Supplementary Fig. S5B). For each subpopulation, PCF is high at small radii, i.e., 10 to 20 μm, indicative of a clustered cell pattern among immediate neighbors. These patterns taper off as the radii increase, i.e., when more cells are considered across wider regions of the tumor. Supplementary Fig. S5C illustrates this visually for a single patient: each subpopulation shows a tendency to group in space within the tissue and does not display a random spatial Poisson distribution (as per random simulation).

To further quantify spatial heterogeneity between subpopulations, we calculated for each cell the percentage deviation (Δ%) of the observed from the expected subpopulation extent (as quantified across whole-tissue available) within the cell's local neighborhood (20 cells). In other words, if cells were distributed randomly in space, the observed abundance of a particular subpopulation in the neighborhood of any given cell would match the overall subpopulation extents measured across a tumor. However, if an over- or underrepresentation of a particular subpopulation occurs in the topological neighborhood of a given cell, this deviation provides a quantitative depiction of local interactions for that cell. Supplementary Fig. S5D demonstrates that each subpopulation (defined here by MYC, BCL2, and BCL6) has a unique pattern of co-occurrence with other subpopulations in terms of the range of Δ% scores in their local neighborhood. This empirical measurement suggests that typically cells of a particular subpopulation cluster with the same cell type (as shown in Supplementary Fig. S5B). There are patterns of heterotypic interaction with one another (Supplementary Fig. S5D, top, e.g., M+2+6− with M+2–6−), or heterotypic segregation (Supplementary Fig. S5D, top, e.g., M+2+6+ with M−2–6+ in the example tumor sample). Such interactions can be empirically established only through spatial investigation and provide a novel and independent feature of tumor heterogeneity that is patient-specific. These interaction patterns can be stable across different regions of the tumor for the same patient, or more rarely, heterogeneous with spatially varying interaction patterns in different tumor regions (Supplementary Figs. S5E and S6). We conclude from these investigations that B-cell subpopulations of different oncogenic coexpression aggregate spatially in a nonrandom manner (likely reflecting clustering due to parent cell–daughter cell relationships or, alternatively, embedding within local microenvironment milieus).

### Cells Coexpressing MYC and BCL2 without BCL6 Confer Poor Survival in DLBCL

**Figure 2.**
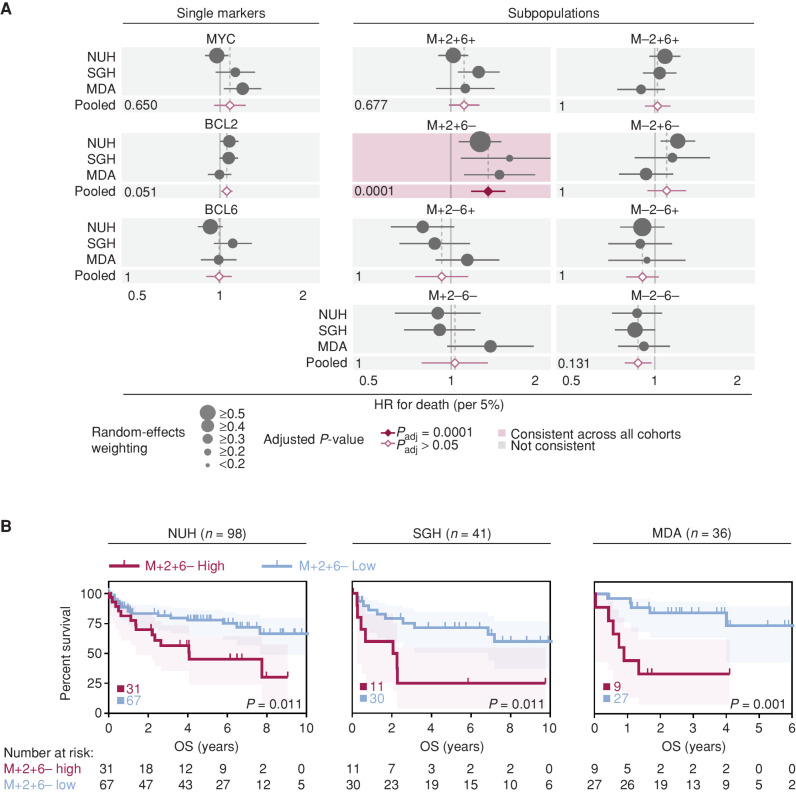
Prognostic significance of subpopulations after R-CHOP therapy. **A,** Pooled univariate Cox PH model analysis for MYC, BCL2, and BCL6 single oncogene and subpopulations percentage extent as predictors of OS across multiple DLBCL cohorts. Percentage extent was used as a continuous variable in the model at 5% increments (see Survival Analysis) for an unbiased comparison between the variables. Pooled *P* values were Bonferroni corrected for single oncogenes and subpopulations independently to adjust for multiple testing and are shown for each variable. Hazard ratio (HR) with 95% confidence interval (CI) per 5%-positivity increment is shown (see also Supplementary Table S4). **B,** Kaplan–Meier OS analysis of dichotomized into M+2+6− high and low groups. Log-rank test, shading denotes 95% CI. An optimal dichotomization cutoff was used for stratification; total patient numbers in each group are shown.

We next evaluated the relationship between MYC/BCL2/BCL6 subpopulations and prognosis, using pretreatment biopsies of R-CHOP (rituximab, cyclophosphamide, doxorubicin, vincristine, and prednisone)–treated DLBCL patients, with clinical data available from three cohorts—National University Hospital, Singapore (NUH, *n* = 98), SGH (*n* = 41), and MDA (*n* = 36). To avoid arbitrary cutoffs, we initially evaluated the percentage of cells with oncogenic combinations as a continuous variable in a univariate Cox proportional hazards (Cox PH) analysis for overall survival (OS). Despite expected variability between cohorts in the prognostic impact of MYC, BCL2, and BCL6 as individual oncogenes (14), the percentage of M+2+6− cells stood out as a consistently poor prognostic variable (Fig. 2A). In this context, we define consistency as when hazard ratios (HR) for death, including 95% confidence intervals, for all cohorts have the same directionality (either consistently greater than 1 or less than 1). The M+2+6− subpopulation showed the greatest effect size and exclusively remained highly statistically significant in a pooled analysis across cohorts (Fig. 2A; Supplementary Table S4). This prognostic association is also illustrated in a dichotomized Kaplan–Meier survival analysis (Fig. 2B). Higher M+2+6− percentage remained statistically significant for poor OS in a multivariate Cox PH model adjusted for clinically relevant DLBCL clinicopathologic parameters of IPI Risk Group and MYC fluorescence *in situ* hybridization (FISH) status (Table 1; Supplementary Table S5). These results suggest that the prognostic impact of these oncogenes in DLBCL is driven by a unique subpopulation of cells expressing MYC and BCL2 without BCL6.

**Table 1.** Multivariate analysis of continuous M+2+6− percentage extent at 5% increments as a predictor of OS in the NUH, SGH, and MDA cohorts of DLBCL (Cox proportional hazards model)

	NUH			SGH			MDA	
	Total cases (*n* = 87) missing values (*n* = 11)			Total cases (*n* = 37) missing values (*n* = 4)			Total cases (*n* = 34) missing values (*n* = 2)	
	HR (95% CI)	*P* value		HR (95% CI)	*P* value		HR (95% CI)	*P* value
Subpopulation								
M+2+6− (continuous, per 5% of extent)	1.3 (1.1–1.6)	0.004		1.6 (1.1–2.3)	0.026		1.6 (1.1–2.3)	0.010
IPI Risk Group		0.402			0.015			0.195
Low	Ref.			Ref.			Ref.	
Intermediate	1.6 (0.63–4.3)	0.314		4.4 (0.88–21.7)	0.299		1.3 (0.19–9.2)	0.780
High	2.0 (0.72–5.6)	0.187		12.6 (2.3–70.1)	0.005		4.5 (0.75–27.1)	0.099
c-MYC translocation status		0.503		—	—			0.536
Negative	Ref.			—	—		Ref.	
Positive	0.61 (0.14–2.6)			—	—		1.6 (0.35–7.7)	

Abbreviations: IPI Risk Group, International Prognostic Index Risk Group; 95% CI, 95% confidence interval; Ref., reference group.

### A Probabilistic Metric Accurately Predicts MYC, BCL2, and BCL6 Coexpression in DLBCL Cells

**Figure 3.**
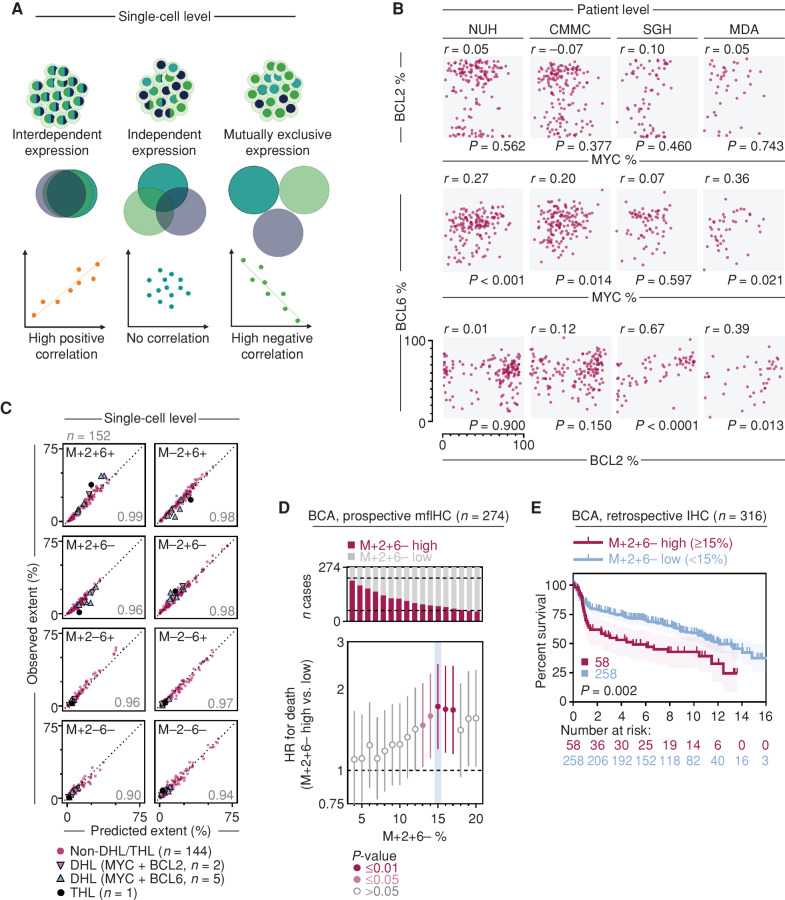
MYC, BCL2, and BCL6 protein coexpression in DLBCL can be inferred from individual marker data. **A,** Schematic of possible relationships between expression of three oncogenes in a population of cells. The distribution of these oncogenes can either reflect interdependent expression, independent/stochastic expression, or mutually exclusive expression. These relationships result in the percentage extent of oncogenes in the tumor being either strongly positively correlated, not correlated, or strongly negatively correlated, respectively. Created with BioRender.com. **B,** Correlation of MYC, BCL2, and BCL6 percentage extent across patients in DLBCL cohorts. Spearman correlation; axes are equivalent in all panels. **C,** Good correlation between probabilistically predicted subpopulation percentage extent based on single oncogene positivity and observed percentage extent in the NUH cohort. Cases of double-hit lymphoma (DHL, MYC+BCL2+ translocations or MYC+BCL6+ translocations) or triple-hit lymphoma (THL) are highlighted. Spearman rho for each correlation is shown; axes are equivalent in all panels. Correlation for other cohorts can be found in Supplementary Fig. S7. **D,** Prospective evaluation of an optimal dichotomization cutoff for M+2+6− percentage extent in the BCA cohort. Univariate Cox PH model at 1% extent positivity increment, HR for death with 95% CI. HR scale is exponential. Optimal dichotomization cutoff is highlighted in blue. **E,** Kaplan–Meier OS analysis of the chromogenic IHC BCA cohort evaluation stratified into M+2+6− metric high and low groups across an absolute value of 15% M+2+6−metric. Log-rank test, shading denotes 95% confidence interval. CMMC, Chi-Mei Medical Center.

As the percentage of MYC+BCL2+BCL6− (M+2+6−) cells correlates with poor survival, we wanted to check if this percentage could be inferred from knowledge of the individual oncogene components. For this, we first describe three possible “scenarios” of oncogene coexpression within a tumor: interdependent expression of each oncogene resulting in overlapping distribution patterns in a population of cells; independent/stochastic expression of each oncogene resulting in random distribution patterns across cells; mutually exclusive expression of each oncogene resulting in spatially excluded distribution patterns (depicted schematically in Fig. 3A). In terms of percentage extent between two oncogenes within a tumor, an interdependent expression would result in a strong positive correlation, an independent/stochastic expression would result in no correlation, and a mutually exclusive expression would result in a strong negative correlation. In contrast to the mutually exclusive expression pattern observed within specific topological compartments in reactive lymphoid tissues, MYC, BCL2, and BCL6 did not show strong correlation or anticorrelation with each other in DLBCL (Fig. 3B).

These results suggest that independent gene regulatory mechanisms drive the expression of *MYC*, *BCL2*, and *BCL6* in DLBCL, and that single-cell coexpression of these oncogenes is largely stochastic. This implies that the percentage of any oncogenic coexpression subpopulation can be inferred by a simple probabilistic metric based on the percentage extent of each component oncogene. If the overall percentages of each component oncogene are known, such a metric describing the percentage of any given subpopulation is derived by multiplying proportions for the presence or absence of each individual oncogene comprising the subpopulation (see Methods). We validated this hypothesis using our single-cell–resolved mfIHC data, observing a highly concordant correlation between observed and predicted percentages for each subpopulation (Fig. 3C; Supplementary Fig. S7).

An extension of this hypothesis is that any quantitative data of MYC, BCL2, and BCL6 allow estimation of the percentage of their coexpressed subpopulations. One such semiquantitative data source of clinical interest is the visual scoring of MYC, BCL2, and BCL6 percentage on chromogenic IHC, which remains the reference method for the assessment of these oncogenes. We checked if our metric could estimate prognostic MYC, BCL2, and BCL6 subpopulations from clinical-grade pathologist scores for chromogenic IHC in a well-characterized cohort of DLBCL from the British Columbia Cancer Agency (BCA; ref. 15). We first performed mfIHC on the BCA cohort to obtain empiric values of the MYC/BCL2/BCL6 coexpressing subpopulations (Supplementary Table S2). M+2+6− percentage extents measured by mfIHC were used to determine an optimal clinically relevant cutoff to classify a patient as a high M+2+6− expressor and therefore likely to have a poor outcome. A dichotomized cutoff of 15% of the M+2+6− subpopulation percentage extent produces the greatest effect size of OS stratification as determined by the Cox PH model between high and low groups in this cohort (Fig. 3D). We then calculated the inferred M+2+6− metric from retrospective semiquantitative chromogenic IHC values. A Kaplan–Meier analysis of the cohort dichotomized into high (≥15% M+2+6− metric) and low (<15%) demonstrated the poor survival of the high metric group (Fig. 3E), confirming the applicability of this probabilistic metric to clinical IHC scoring in DLBCL.

One key consideration for applicability of this metric as a biomarker would be the size of the region to be sampled for adequate representation. As our cohorts were studied in tissue microarray format with small (1 mm) cores, we also evaluated our multiplexed analysis on whole-tissue DLBCL tumor sections (*n* = 8; UP; Supplementary Table S2). The variance in M+2+6− percentage extent in different tissue regions/image fields was low (Supplementary Fig. S8A). Importantly, with the low variance, sampling just two or more high-power diagnostic fields is generally reliable to determine M+2+6− high vs. low samples using a single threshold cutoff of 15% (Supplementary Fig. S8B). Overall, these findings speak to the possible clinical applicability of the M+2+6− metric for pathologist scored chromogenic IHC, which requires validation in future prospective studies.

### Estimation of MYC, BCL2, and BCL6 Coexpressing Subpopulations Can Be Extended to Gene-Expression Data

**Figure 4.**
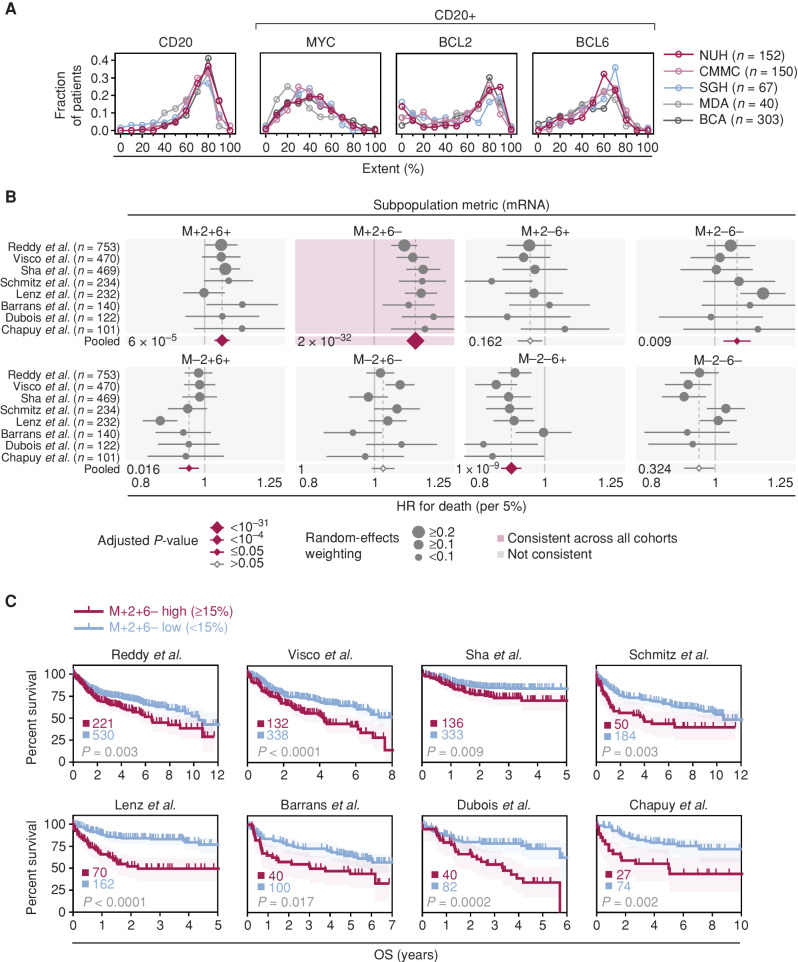
Validation of prognostic significance of the M+2+6− subpopulation metric in gene-expression datasets. **A,** Distribution of single oncogene positivity in DLBCL cohorts as assessed by mfIHC (see Supplementary Fig. S9A and S9B). **B,** Impact of subpopulation metrics in GEP datasets on OS. Pooled univariate Cox PH model analysis; metric was used as a continuous variable in the model at 5% increments. HR per 5% increment with 95% CI is shown; CI are proportional on both tails but are capped at the graph's edges. Pooled *P* values were Bonferroni corrected to adjust for multiple testing and are shown for each subpopulation. **C,** Kaplan–Meier OS analysis of GEP cohorts stratified uniformly across absolute 15% M+2+6− metric into -high and -low groups. Log-rank test, shading denotes 95% CI. Total patient numbers in each group are shown. CMMC, Chi-Mei Medical Center.

We hypothesized that if the percentage of MYC/BCL2/BCL6 subpopulations could be inferred from individual oncogene components on IHC, then our metric could also be computed from other quantitative data measuring MYC/BCL2/BCL6 expression. This could also extend to gene-expression profiling (GEP) with the assumption of positive mRNA-protein correlation (which has been reported for MYC and BCL2 expression in DLBCL; ref. 16). To transform gene-expression data into predicted percentage extents, we first established an empirical cumulative distribution function (eCDF) for each individual protein marker (MYC/BCL2/BCL6 percentage extents) across five mfIHC cohorts (*n* = 712). Importantly, the eCDFs of single-marker protein and subpopulation percentages are similar across all five mfIHC cohorts of patients (Fig. 4A), allowing compilation of an aggregated consensus protein distribution for each oncogene (Supplementary Fig. S9A and S9B). We then perform eCDF mapping (matching percentile points in the eCDF of mRNA measurements to those in the eCDF of the corresponding protein scores) and convert the oncogene's quantitative mRNA score into an inferred single-marker percentage extent (see Methods; Supplementary Fig. S9C). These inferred percentage extents from mRNA data could be used to generate our aforementioned metric, estimating proportions of subpopulations (Supplementary Table S6).

We then utilized GEP cohorts with available survival data after R-CHOP treatment (8 cohorts, *n* = 2,521) to evaluate the prognostic impact of the RNA-based metric for M+2+6− prediction. The M+2+6− metric remained the only RNA-inferred subpopulation consistently associated with poor survival across eight distinct cohorts of DLBCL patients (Fig. 4B; Supplementary Table S7). As with the mfIHC-based results, the pooled analysis revealed that the M+2+6− metric had the greatest effect size and most statistically significant *P* value with respect to HR for death. In the GEP analysis, metrics representing other subpopulations did show occasional statistically significant survival associations—but these were not consistent, of a smaller effect size and by many orders of magnitude less statistically significant compared with the M+2+6− metric. The M+2+6− metric was consistently prognostic in both microarray gene-expression–based cohorts (17–22) and RNA sequencing (RNA-seq)–based cohorts (23, 24), attesting to its validity for mRNA quantified from varying platforms. The significance of the M+2+6− metric was further corroborated in a multivariate Cox PH analysis correcting for IPI Risk Group and cell-of-origin (COO) gene-expression signature, where M+2+6− remained a statistically significant predictor of poor survival in seven out of eight cohorts as a continuous variable (Supplementary Table S8).

Finally, we performed an independent study on the mRNA-based M+2+6− metric in samples with biomarker data available from the GOYA clinical trial cohort (25). GOYA was a randomized phase III trial (NCT01287741) comparing two different anti-CD20 antibodies (rituximab and obinutuzumab) in combination with CHOP chemotherapy. Although the trial did not show differences in survival between the two arms, it remains a valuable source of evaluating molecular determinants of survival in chemoimmunotherapy-treated DLBCL. Although BCL6 IHC was not available for the GOYA samples, MYC and BCL2 IHC scores showed statistically significant correlations with mRNA levels of MYC and BCL2, respectively, supporting the rationale for the extension of our metric from protein to mRNA (Supplementary Fig. S10A and S10B). The M+2+6− metric was associated with progression-free survival and OS in this dataset, in both univariate and multivariate analyses (Supplementary Table S9). Finally, Kaplan–Meier OS analysis on GOYA as well as other publicly available GEP datasets confirmed that our previously established 15% threshold cutoff was relevant for prognostic stratification (Fig. 4C; Supplementary Fig. S10C; Supplementary Table S10).

### Molecular Characteristics of M+2+6− High DLBCL

**Figure 5.**
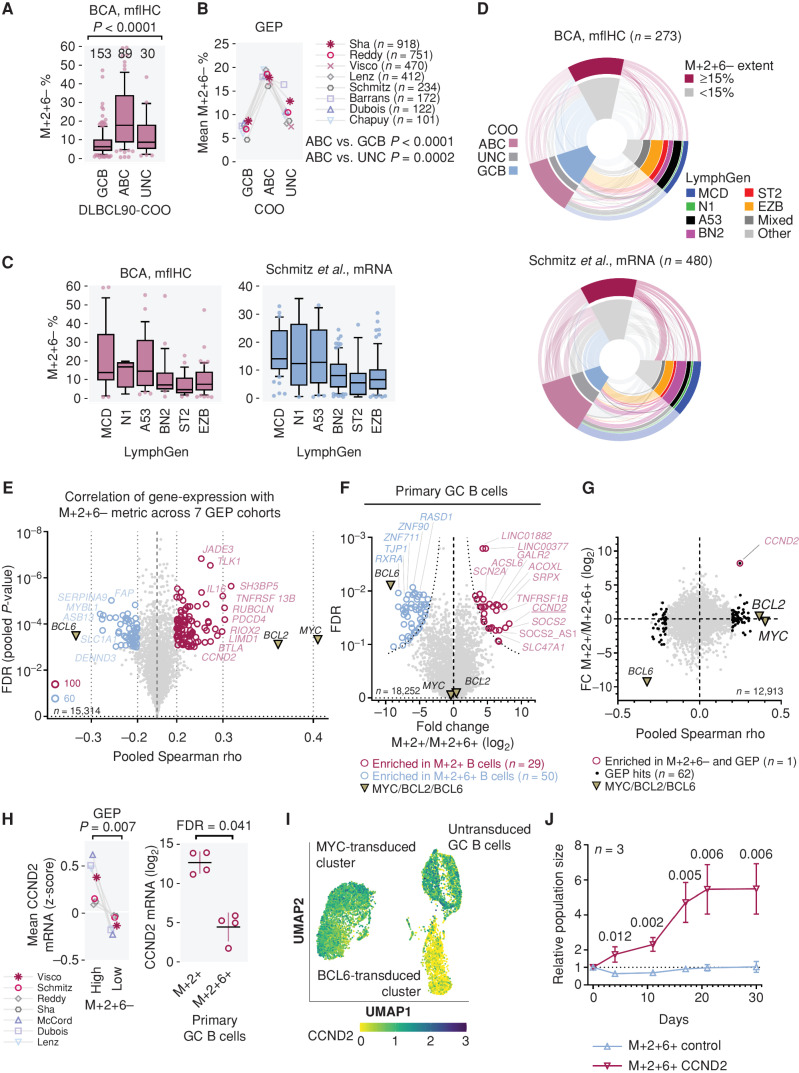
Transcriptomic analysis of M+2+6− high cases and potential role of *CCND2*. **A,** Correlation of observed M+2+6− percentage extent in the BCA cohort with the cell-of-origin (COO) DLBCL90-COO signature. Bonferroni corrected Kruskal–Wallis test for ABC vs. others. **B,** Correlation of M+2+6− metric in GEP cohorts with COO signatures. Mean M+2+6− metric value per group per cohort is shown. Bonferroni corrected paired-samples *t* test. **C,** Correlation of the M+2+6− percentage extent and metric evaluated by mfIHC and mRNA inference, respectively, with genetic subtypes (LymphGen classification). **D,** Sankey plot of M+2+6− dichotomized grouping matched with molecular features. **E,** Volcano plot of pooled direct correlation of gene mRNA expression and M+2+6− metric across seven GEP cohorts. Genes highly correlated with M+2+6− metric across datasets at absolute Spearman rho ≥0.2 and FDR≤0.001 are shown (see also Supplementary Table S11). The abscissa is scaled exponentially. **F,** Differential gene expression between primary GC B cells overexpressing M+2+ and M+2+6+ (see also Supplementary Table S12). Analysis is generated from 4 biological replicates from each condition, from cells of independent donors. **G,** Genes highly enriched in M+2+6− cells: correlation of results from **E** and **F**. **H,***CCDN2* gene expression in GEP cohorts in patients dichotomized by M+2+6− 15% metric (left) and in primary B cells (right). Paired *t* test (left); mean with standard deviation and FDR (FDR as per Supplementary Table S12) for *t* test (right). **I,** Single-cell RNA-seq of GC primary B cells transduced either with BCL2 and MYC (MYC-transduced) or BCL2 and BCL6 (BCL6-transduced). Untransduced GC primary B cells are also included. Expression of *CCND2* is indicated in color. **J,** Proliferation analysis of M+2+6+ primary GC B cells overexpressing cyclin D2 (*CCND2*) compared with M+2+6+ primary GC B cells transduced with an empty vector (EV). Analysis performed with 3 biological replicates for each condition, using cells from 3 independent patients; mean with standard deviation; *t* test. UMAP, Uniform Manifold Approximation and Projection.

Inference of oncogene coexpression from GEP datasets allows an extended avenue for comparative analysis with other molecular characteristics in DLBCL, which can be utilized to describe molecular features characterizing the M+2+6− subpopulation. We first investigated the relationship between (mfIHC-generated) M+2+6− percentage extent and GEP-determined COO data available for the BCA cohort. M+2+6− percentage extent was associated with the ABC COO subtype (Fig. 5A), and this association with ABC COO was consistent for the inferred M+2+6− metric across the GEP datasets (Fig. 5B). The M+2+6− subpopulation and M+2+6− metric also was associated with the unfavorable MCD and A53 genetic subtypes of DLBCL (Fig. 5C; ref. 26). These relationships are depicted categorically in an integrated fashion in Fig. 5D.

To derive single-gene associations with the M+2+6− subpopulation on the bulk level, we correlated the M+2+6− metric with gene expression across seven GEP cohorts (*n* = 3,180 samples, Fig. 5E; Supplementary Table S11). One-hundred sixty genes consistently correlated either positively or negatively with the M+2+6− metric (Fig. 5E). To narrow down those of key biological significance in the first instance, we leveraged on the observation that M+2+6− percentages are strongly correlated with a poor prognosis, whereas the survival association with M+2+6+ is much weaker. We compared gene expression of DLBCL with gene expression of primary human tonsil-derived GC B cells immortalized by either the overexpression of *MYC* and *BCL2* (M+2+6−) or *MYC*, *BCL2*, and *BCL6* (M+2+6+; ref. 27). Because BCL6 is a transcriptional repressor (28), we hypothesized that the absence of BCL6 could influence the transcriptional profile of the M+2+6− subpopulation (Fig. 5F; Supplementary Table S12). Cross-comparing genes enriched in the M+2+6− GC B cells with genes correlated with the M+2+6− population from bulk clinical GEP datasets, we found that *CCND2* (which codes for cyclin D2) was highly enriched in both groups (Fig. 5G and H). Furthermore, single-cell RNA-seq (scRNA-seq) of a tonsil-derived GC B-cell sample clearly demonstrated an inverse correlation between *CCND2* and *BCL6* expression (Fig. 5I), confirming prior observations that *CCND2* is transcriptionally repressed by BCL6 (29, 30). We then transduced *CCND2* in M+2+6+ immortalized B cells, which were characterized by low background levels of cyclin D2 expression (Supplementary Fig. S11). The transduced M+2+6+/*CCND2*^High^ population started as a relatively small fraction, but rapidly expanded over time eventually outgrowing the M+2+6+/*CCND2*^Low^ population (Fig. 5J; Supplementary Fig. S11), confirming that increased cyclin D2 expression can confer a fitness advantage to cells with MYC and BCL2. Cyclin D2 expression has been reported as a marker for an adverse outcome in DLBCL (31, 32).

### Single-Cell Transcriptomic Analyses of M+2+6− Cells in DLBCL

**Figure 6.**
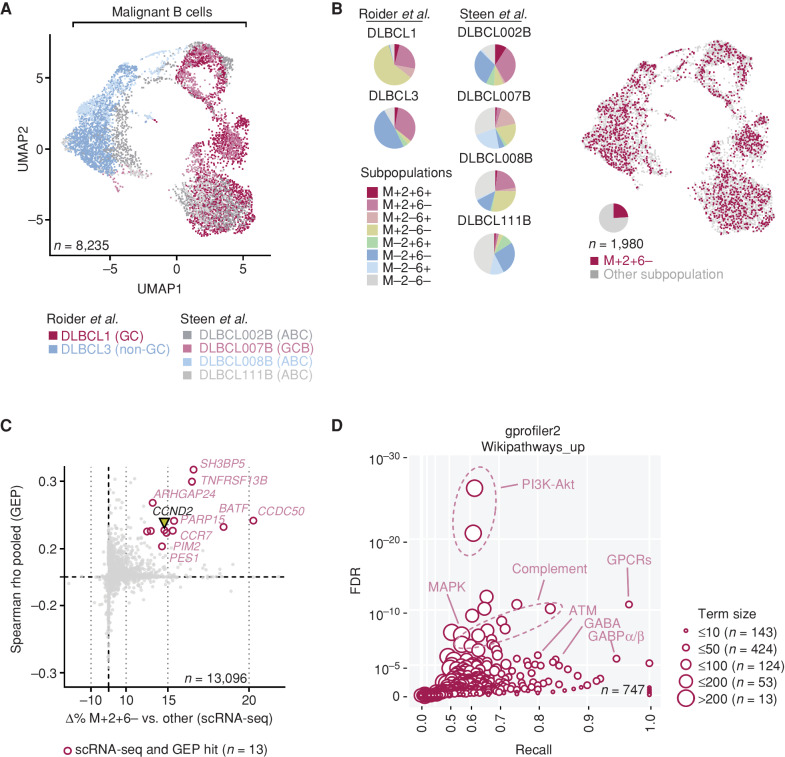
Evaluation of M+2+6− cells in scRNA-seq datasets of DLBCL. **A,** Uniform Manifold Approximation and Projection (UMAP) of malignant B cells from the Roider and Steen cohorts. **B,** Proportion of subpopulations across samples and annotation of M+2+6− cells in UMAP. **C,** Correlation of genes enriched in the M+2+6− subpopulation as evaluated by scRNA-seq with hits from the bulk GEP cohorts (Fig. 5E). *CCND2* is highlighted and is among the concordant hits (see also Supplementary Table S13). **D,** WikiPathways terms enrichment among genes positively associated with M+2+6− cells. Both axes in **C** and **D** are scaled exponentially for clarity (see also Supplementary Table S14).

To further understand other molecular determinants underlying poor prognosis in cases with high numbers of M+2+6− cells, we leveraged on scRNA-seq datasets to profile the transcriptomic characteristics of M+2+6− malignant B cells within DLBCL samples. We first harmonized single-cell transcriptomic data from 6 DLBCL patient samples from two independent datasets (refs. 33, 34; Fig. 6A). Figure 6B demonstrates that the M+2+6− subpopulation is well represented in all samples. We identified genes associated with the M+2+6− B-cell subpopulation (Fig. 6C) and confirmed *CCND2* expression being more abundant in M+2+6− B cells compared with all other malignant B cells. In total, 13 concordant genes were enriched in both the scRNA-seq data and the bulk RNA-seq data (Supplementary Table S13). These include ABC-DLBCL–related genes such as the ROCK1 target PES1, which intersects MYD88 and NF-κB signaling (35), the PIM2 kinase whose overexpression has been associated with unfavorable DLBCL biology (36), and the IRF4 interactor BATF (37). Finally, Fig. 6D depicts a pathway analysis on this single-cell–resolved transcriptomic data revealing that the PI3K–AKT pathway, immune responses (including the complement pathway), as well as G-protein receptor–coupled signaling, were among the significantly enriched terms in M+2+6− B cells compared with all other malignant B cells (Fig. 6D; Supplementary Table S14). The enrichment of PI3K–AKT signaling signatures is particularly intriguing, as inhibitors of this pathway (e.g., copanlisib) are clinically applicable, suggesting a possible therapeutic strategy for unfavorable M+2+6− high tumors. Additional mechanistic studies will be needed to understand the relative significance and interplay between these genes and pathways in conferring poor outcome in M+2+6−. Of general significance, however, these results illustrate how the estimation of oncogene coexpression phenotypes through gene-expression data, coupled with single-cell resolved transcriptomic data, may uncover novel biological insight.

## DISCUSSION

In this paper, we show for the first time that subpopulations of tumor cells expressing combinations of oncogenes at the single-cell level influence patient prognosis. We also show that (under conditions of independently regulated expression), these subpopulations can be inferred from quantitative single oncogene expression data, generating a metric that has a remarkable concordance to actual observed single-cell coexpression on multiplex IHC. We show two applications of predicting oncogenic subpopulations in the setting of DLBCL. First, the M+2+6− metric can be generated from diagnostic IHC scores, offering a refined method for utilizing MYC, BCL2, and BCL6 expression for prognostic use in DLBCL. Secondly, by estimating subpopulation percentages from GEP datasets, we demonstrate the feasibility of identifying molecular features associated with a poor prognostic oncogene combination from the plethora of gene-expression studies available for a given disease. Such features could identify therapeutic targets or offer biological insight–as with our demonstration that the cell-cycle regulator cyclin D2 (*CCND2)* may play a role in the aggressive phenotype of M+2+6− cells. Cyclin D2 promotes the G_1_–S transition of hematopoietic cells (38), enhances cytokine induced-proliferation (39), and is stabilized by EBV infection (40), highlighting the rationale for further studies of *CCND2* in DLBCL pathogenesis and evolution.

Our single-cell–resolved quantitative imaging confirms that ITH in coexpression of MYC, BCL2, and BCL6 exists in almost every case of DLBCL. This coexpression shows distinct spatial organization with nonrandom clustered patterns, supporting the concept that forces beyond genetic heterogeneity shape DLBCL evolution. These findings also suggest that quantitative assessment of the M+2+6− subpopulation potentially refines the MYC-BCL2 “double expressor lymphoma” (DEL), a term used to describe DLBCL with overexpression of MYC and BCL2 protein in the absence of underlying genetic rearrangements (7, 41, 42). DEL is typically defined as >40% MYC-positive cells and >50% BCL2-positive cells (measured independently). As these DEL classifications do not take DLBCL ITH (43, 44) into account, it was not known if DELs represent two distinct and coexisting clonal phenotypes within a lymphoma—one expressing MYC and the other BCL2. Nor was it understood why the poor outcome of DEL is exacerbated when BCL6 expression is absent (9, 45). These issues are addressed by the description of the M+2+6− subpopulation, which describes the phenomenon at single-cell resolution. DEL remains relevant in the era of genetic DLBCL classification (46) and novel targeted therapies. For example, patients with the DEL phenotype show improved survival on the polatuzumab arm in comparison with the R-CHOP arm of the POLARIX trial (47). Additional studies are required to clarify the relevance of the M+2+6− percentage extent in this setting.

Lymphomas that harbor translocations in MYC, BCL2, and/or BCL6, termed double-hit or triple-hit lymphomas (DHL/THL; ref. 48), have a particularly poor outcome (49–52). Recently, prognostic gene-expression signatures have been developed that accurately classify such DHL or THL cases: double-hit signature (DHITsig; ref. 49) and molecular high-grade (MHG; ref. 17). Of DLBCL that are DHITsig positive, the majority fall within the EZB genetic subtype (26). Using our single-cell–resolved approach to DEL, we saw that cases with higher M+2+6− metrics were typically assigned to either the A53 or MCD genetic subtype (Fig. 5C). These results are consistent with double-hit (and DHL-like/MHG) lymphoma being biologically distinct from DEL, though both types display poor prognoses. Figure 5 demonstrates an association between the M+2+6− subpopulation and the ABC COO subtype as well as the MCD genetic subtype, consistent with observations that the MCD subtype is almost exclusively ABC (26). However, the M+2+6− subpopulation also shows enrichment in both A53 and “other” unclassified genetic subsets, and we posit that distinct genetic backgrounds can converge on this final phenotype through distinct mechanisms.

This is a proof-of-concept study with some limitations. First, the prognostic impact of M+2+6− percentages derive from retrospective analyses and need prospective validation. In this article, we have, where possible, presented HRs as a continuous variable, ascribing a risk score per unit of measure (per 5% of M+2+6− percentage extent). This is an unbiased method by which to assess the risk of the M+2+6− subpopulation, however, a more pragmatic approach for clinical biomarker use is to develop a standardized cutoff for the M+2+6− percentage. Our initial analyses from the BCA cohort and GEP datasets (including the GOYA trial) suggest that ≥15% M+2+6− percentage extent may be a suitable starting point for such prospective validation studies. Second, the prevalence of staining artifacts within FFPE tissue samples required us to use a semiautomated method (manual checking of intensity threshold per image), which is not optimal for upscaling of this approach. The development of deep learning approaches refined for evaluating marker positivity based on fluorescence intensity but also considering other background/morphologic features would be key toward implementing this diagnostic method into the clinic. Finally, although we use threshold-based positivity scoring in this study, per-cell quantitative proteomic data (ideally obtained through an amplification-free method such as imaging mass spectrometry) is an understudied area that may yield even greater resolution toward assessing prognostic outcomes.

The probabilistic metric we describe, which predicts oncogenic coexpression, holds true only when the expression of the oncogenes is independently regulated and will need validation in the setting of other oncogenes/cancers. Nonetheless, our demonstration that the M+2+6− phenotype confers poor survival in four empirically evaluated cohorts (mfIHC) and nine inferred cohorts (GEP) of DLBCL underscores the clinical importance of evaluating the ITH generated by the coexpression of oncogenes and suggests that similar studies in other cancer types will be informative. Oncogene ITH occurs at multiple molecular levels in cancer: genetic (53, 54), epigenetic, transcriptomic, and proteomic (55), and affects clinical phenotypes (1, 56). Single-cell approaches to evaluate genetic (57), transcriptomic (58, 59), and proteomic ITH have provided valuable insight into microevolutionary processes operating in cancer (60, 61). However, due to high experimental costs, the number of patients represented in scRNA-seq and mass cytometry datasets are invariably small, thus precluding clinically meaningful multivariate analyses. Here we demonstrate that multiplexed microscopy through mfIHC, though limited in multiplexing potential compared with scRNA-seq, is well suited to measure the clinical impact of single-cell–resolved ITH in clinically annotated patient cohorts.

## METHODS

### Samples and Datasets

Tonsils (*n* = 15) from patients diagnosed with chronic tonsillitis, reactive lymph nodes (*n* = 2), and DLBCL [*n* = 152, tissue microarray (TMA) format] were obtained from the NUH [approved by the Singapore NHG Domain Specific Review Board B study protocol (2015/00176)]. Additional DLBCL TMAs for quantitative mfIHC analyses were from the CMMC cohort (*n* = 150), the SGH cohort (*n* = 67), and the MDA cohort (*n* = 40). Pretreatment biopsies of the NUH, SGH, and MDA cohorts were used for survival analysis following standard first-line R-CHOP-like therapy. A TMA from the BCA cohort (*n* = 274) with first-line R-CHOP-like follow-up data was used as a validation cohort (49). Eight whole-slide DLBCL sections retrieved from the archives of the Tumor Immunology Laboratory of the University of Palermo were included in the study as approved by the University of Palermo Institutional Review Board (IRB) 09/2018. Full patient characteristics for all the above cohorts are provided in Supplementary Table S15. Samples from all institutions were obtained through IRB-approved ethics protocols, with written informed consent from the patients, or with IRB-approved waivers of consent where applicable in accordance with the ethical guidelines of the Declaration of Helsinki. Material transfer agreements from all providing institutions were incorporated into the framework of an NUS IRB-approved translational study (H-19-055E). Preprocessed gene-expression data were obtained from Gene Expression Omnibus (GEO; RRID: SCR_005012; ref. 62) for datasets GSE117556 (*n* = 928; ref. 17), GSE125966 (*n* = 553; ref. 63), GSE31312 (*n* = 498; ref. 18), GSE10846 (*n* = 420; ref. 19), GSE87371 (*n* = 221; ref. 20), GSE32918 (*n* = 172; ref. 21), and GSE98588 (*n* = 137; ref. 22). Raw gene-expression data for Reddy and colleagues (*n* = 775; ref. 23) were obtained through The European Genome-phenome Archive (EGA; https://ega-archive.org/) at the European Bioinformatics Institute, Study ID: EGAS00001002606. Raw gene-expression data from Schmitz and colleagues (*n* = 481; ref. 24) were obtained from the NIH database of Genotypes and Phenotypes (dbGaP; RRID:SCR_002709), accession number: phs001444.v2.p1; The Genomic Variation in Diffuse Large B-Cell Lymphomas study was supported by the Intramural Research Program of the National Cancer Institute, NIH, Department of Health and Human Services. Clinical data associated with the GOYA dataset (GSE125966) were analyzed in collaboration with F. Hoffmann-La Roche Ltd.

### Quantitative mfIHC and Scoring

Quantitative mfIHC was performed using sequential OPAL-TSA staining as described in detail previously (ref. 64; Supplementary Tables S16 and S17). Images were acquired using the Vectra 2 imager and analyzed using inForm2.4.8 (RRID: SCR_019155). DAPI nuclear staining and CD20 membrane staining were used to segment cells. The mean membrane intensity per cell was captured for CD20; the mean cytoplasm intensity per cell for BCL2; and the mean nuclear intensity per cell for both MYC and BCL6. For each image, cells with a marker intensity above a given intensity threshold for that image were declared to be positive for that marker. A pathologist manually inspected each image to determine a reliable threshold for each marker that resulted in minimal false-positive and false-negative assignments. Images were examined in pseudocolor brightfield. All cohorts were evaluated in a tissue microarray (TMA) format; depending on tissue availability, between 1 and 9 high-power 700 × 500 μm imaging fields were captured and evaluated per patient in the NUH cohort, and two 1,400 × 1,000 μm fields were evaluated in the CMMC, SGH, MDA, and BCA cohorts. For each of the DLBCL whole-tissue sections, 5 to 8 700 × 500 μm imaging fields were evaluated. Once positivity thresholds were set for each marker per image, the quantitative image data (mean intensity per cell and the intensity threshold per marker for each image) were exported to calculate per-cell marker positivity and coexpression status. Subsequently, the percentage of cells within the CD20^+^ B-cell compartment that were ascribed a given subpopulation (M+2+6+, M+2+6−, M+2−6+, M+2−6−, M-2+6+, M−2+6−, M−2–6+, and M−2–6−) were calculated for each patient. Scores from patients with multiple cores were a mean across all cores, weighted on cell number per core.

### Survival Analysis

For unbiased survival associations, subpopulations percentage extents were evaluated as continuous variables in Cox PH models at 5% unit increments (albeit 0%–1% compromising the first unit, followed by 1%–5%, 5%–10%, etc.). HRs are displayed per unit (of 5% extent). Variables satisfied proportional hazards assumptions. To leverage on the multicohort design of this study, associations of each subpopulation extent were evaluated in a univariate model in individual cohorts, which was followed by effect size and *P* value pooling. Effect sizes were pooled by a random-effects model to mitigate interstudy heterogeneity, and Paule–Mandel heterogeneity variance estimator was applied due to the small number of cohorts. The pooled *P* values were adjusted for multiple hypothesis testing using Bonferroni correction (8 hypotheses). Tests were performed using the R “survival” package (RRID: SCR_021137) and pooled using the R “poolr” package. Multivariate Cox PH models were performed in SPSS 23 (RRID: SCR_002865). For Kaplan–Meier analyses, a log-rank test was performed using GraphPad Prism 9 (RRID: SCR_000306). Cohorts were dichotomized at an optimal cutoff in exploratory analyses, and subsequently, analyses were dichotomized at an established positivity threshold of ≥15% of M+2+6− extent (actual or using the metric). Statistical tests were two-sided and *P* ≤ 0.05 was considered statistically significant.

### Probabilistic Inference of Colocalization

Assuming the independent distribution of positivity between MYC, BCL2, and BCL6, a probability-based algorithm using single oncogene scores was derived to predict the percentage extent of subpopulations, i.e., permutations of MYC, BCL2, BCL6-positivity and -negativity in CD20^+^ cells in DLBCL samples:




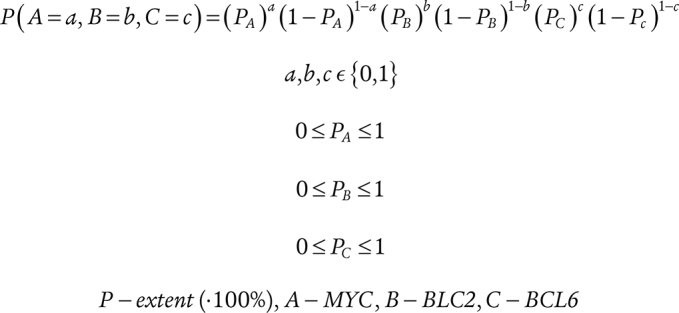




### Mapping of mRNA Expression Data into Percentage Extent

Transformation of MYC, BCL2, and BCL6 mRNA levels into predicted percentage extent values requires an initial transformation step to map percentile points from RNA data onto protein data distributions, similar in principle to QQ plots where data are transformed into equivalent Gaussian data. For each protein marker aggregated across five mfIHC cohorts (*n* = 712), the empirical cumulative distribution function (eCDF) of mfIHC-based MYC/BCL2/BCL6 percentage extents was estimated as the benchmark distribution. With a biological assumption that mRNA expression is correlated with protein percentage scores for these oncogenes (16), we perform eCDF mapping (matching percentile points in the eCDF of mRNA measurements to those in the eCDF of the corresponding protein percentage scores), and then convert the quantitative mRNA score into an inferred single-marker percentage extent from the mRNA mapped CDF (mCDF). Subpopulation extents are subsequently inferred from the mapped single-marker mRNA values using the probabilistic cellular co-occurrence assumption.

To this end, eCDF of the corresponding mRNA expression levels was obtained from the individual subjects in the external datasets. Both eCDFs, mfIHC and mRNA, were smoothed by a Gaussian kernel smoother with a bandwidth parameter set at 1% of the entire range, and the percentile points on the smoothed CDFs were matched between the two datasets. Because eCDF is a monotone increasing function, this operation guarantees one-to-one mapping between the two eCDFs, and we used this map to translate the mRNA measurements into the approximate protein percentages across the individual subjects in the external dataset. The mapping procedure is performed independently for each mRNA dataset with the consensus protein eCDF to mitigate batch effects between datasets that are created through different technologies, and also thus retaining the mRNA cohorts as independent datasets.

The mapping source code for this approximation is available at GitHub (RRID: SCR_002630): https://github.com/MichalMarekHoppe/Patterns-of-oncogene-co-expression-at-single-cell-resolution-influence-survival-in-lymphoma.

### Correlation of Gene Expression and M+2+6− Metric

Preprocessed gene-expression matrices submitted by the original authors were used for microarray-based datasets (Sha and colleagues, ref. 17; McCord and colleagues, ref. 63; Visco and colleagues, ref. 18; Lenz and colleagues, ref. 19; and Dubois and colleagues, ref. 20) and RNA-seq datasets were processed in-house (Reddy and colleagues, ref. 23; Schmitz and colleagues, ref. 24). Analyses were done for a consensus of 15,314 genes annotated in-house. Barrans and colleagues (21) and Chapuy and colleagues (22) data were not evaluated in exploratory analyses due to a lower number of genes in the original mapping and lower number of samples. Standardized gene expression was correlated with the M+2+6− metric (Spearman correlation) − Spearman rho with 95% confidence intervals was obtained using the R “DescTools” package and results were pooled using the R “poolr” package. Genes with a pooled Spearman rho value of ≥0.2 and a false discovery rate (FDR) ≤0.001 were considered hits in this analysis.

### Differential Gene Expression in Primary GC B Cells

Total RNA was isolated from primary transduced B cells using the TRIzol extraction method. Insert cDNA library creation (250–300 bp eukaryotic mRNA) and standard polyA paired-end sequencing on Illumina Hiseq-4000 (RRID: SCR_016386) PE150 was performed by NovogeneAIT. Raw sequencing files were processed using standard pipelines available publicly on the CSI NGS Portal (65). Gene expression of 18,252 genes was compared between four transduced GC B cells samples of M+2+6+ and four M+2+6− samples from independent donors (see “Generation of immortalized patient-derived GC B cells, and *CCDN2* analysis” section for details on GC B-cell transduction and refs. 27 and 66). FDR of a two-sided *t* test was used to define differently expressed genes. The R package “stats” was used to perform the *t* test. Hits were defined by meeting an arbitrary dynamic threshold criterion defined by the rational function (see the dashed line Fig. 5F):




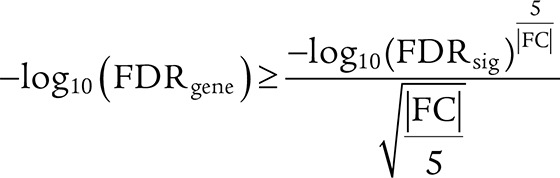




where ${\mathrm{FD}}{{\mathrm{R}}_{{\mathrm{gene}}}}$ is the FDR value of the gene tested, ${\mathrm{FD}}{{\mathrm{R}}_{{\mathrm{sig}}}}$ is an arbitrary threshold of significance of 0.05, and $\;|{\mathrm{FC}}|$ is the absolute value of ${\mathrm{lo}}{{\mathrm{g}}_2}$ fold change difference between mean expression in M+2+6− and M+2+6+ samples.

### Generation of Immortalized Patient-Derived GC B Cells, and *CCDN2* Analysis

Discarded tonsil tissue was collected after tonsillectomy at Addenbrooke's ENT Department, Cambridge, UK, with written informed consent from the patient's parent/guardian. Ethical approval for human tissue use was granted by the Health Research Authority Cambridgeshire Research Ethics Committee (REC no. 07/MRE05/44). Human primary GC B cells were isolated from fresh tonsils with Human B-cell Negative Selection Isolation Kit II (MACS, Miltenyi Biotec, cat. no. 130-091-151) supplemented with anti-IgD and anti-CD44 antibodies as described previously (27, 66). GC B cells were frozen immediately after isolation. Tissue from two female and one male donor ages 4 to 5 years was collected in September 2018. As these were primary cells, authentication and *Mycoplasma* testing were not performed.

After thawing, cells were cultured *in vitro* on irradiated YK6-CD40lg-IL21 follicular dendritic feeder cells in Advanced RPMI-1640 (Invitrogen, cat. no. 12633020) supplemented with 20% Gibco FCS (Thermo Fisher Scientific, cat. no. 10270-106) and 1× Gibco penicillin–streptomycin–glutamine (from 100×, Thermo Fisher Scientific, cat. no. 10378016) as previously described (27, 66). GC B cells were passaged 1 to 3 times before they were stably transduced with BCL6-T2A-BCL2 (27) and MYC-IRES-GFP retrovirus according to the protocol described previously (27, 66). *MYC* was cloned into the MSCV-IRES-GFP plasmid (RRID: Addgene_20672) to create the MSCV-MYC-IRES-GFP construct expressing MYC and GFP. The pBMN-IRES-Lyt2 (EV) retroviral vector was a kind gift from Dr. Louis Staudt, National Cancer Institute, USA; *CCND2* was cloned into pBMN-IRES-Lyt2 to create the pBMN-CCND2-IRES-Lyt2 construct. Subsequently, cells were stably transduced with either EV (pBMN-IRES-Lyt2) or CCND2-IRES-Lyt2 lentivirus. The live-cell fractions of EV or *CCND2*-transduced cells were assessed by flow cytometry after staining for Lyt2 with antimurine CD8a-APC antibody (Miltenyi Biotec; cat. # 130-117-776, RRID: AB_2728039) and observed for 30 days. GFP protein was quantified by flow cytometry as a proxy for MYC expression.

### Analysis of *CCND2* in scRNA-seq Data

For scRNA-seq experiments, primary human GC B cells from a single donor were transduced with BCL2 and BCL6, or BCL2 and MYC (see “Generation of immortalized patient-derived GC B cells, and *CCDN2* analysis” section for transduction details and refs. 27 and 66). Seven days after transduction, cells were pooled and subjected to scRNA-seq using the 10X Genomics platform. Fresh transduced GC B cells from the same donor were spiked into the sequencing reaction. Raw fastq files were processed using cellranger (v3.1.0); the alignment was performed against the GRCh38-3.0.0 version of the *Homo sapiens* reference genome; the quantification and filtering of cells were done using default parameters.

Further filtering applied on the expression matrix was based on upper and lower bounds on the distributions of counts and features, and on the proportions of reads incident to mitochondrial DNA (mt%) and ribosomal genes (rp%). Cells with values outside these ranges (counts per cell/sequencing depth >5,000, number of features <2,000 or >8,000, mt%>15% rp%>50%) were considered outliers and excluded from downstream analyses. Post filtering, mitochondrial and ribosomal genes were excluded from the expression matrix. The expression matrix was log-normalized using the NormalizeData function in the Seurat package (v3.2.2; ref. 67).

Dimensionality reductions [PCA followed by Uniform Manifold Approximation and Projection (UMAP)], as well as clustering (Louvain method) were conducted in Seurat; the optimal number of clusters was selected based on default clustering parameters. Following an assessment of the stability of clustering results, for the subsequent steps, we focused on the 2,000 most abundant genes, determined across all cells in the dataset. Marker genes, determined for each cluster against all other genes, were identified based on differential expression tests (in Seurat) i.e., genes with log_2_(FC) >0.25, and adjusted *P* values, under a Benjamini–Hochberg multiple testing correction, less than 0.05. The data were also made available as a Shiny app (RRID: SCR_022756; ref. 68) at the link: https://bioinf.stemcells.cam.ac.uk/shiny/hodson/MYC-BCL2-BCL6_project.

### Reprocessing of scRNA-seq Datasets

In this study, six DLBCL samples from two publicly available DLBCL scRNA-seq datasets were utilized. Dataset GSE182434 (33), containing sample pairs of B cells and non B cells for 3 ABC-DLBCL tumors and 1 GCB DLBCL tumor, was downloaded from the GEO database. B-cell samples were provided with annotations containing cell type and condition (i.e., tumor or normal). Only cells annotated as tumor and B cells were used for the analysis. DLBCL scRNA-seq dataset generated by Roider and colleagues (34) was downloaded from the heiDATA database (https://heidata.uni-heidelberg.de) from the link https://doi.org/10.11588/data/VRJUNV. The dataset contained four GC-derived DLBCLs, two of which were transformed follicular lymph nodes, which were excluded from the analysis and one nongerminal center-derived DLBCL. Upon further examination of the shared nearest neighbor clusters of the samples (original paper, Fig. 3B; ref. 34), one of the GC-derived DLBCL samples clustered closely with the transformed follicular lymph node cluster and was hence excluded from the analysis. Samples were provided with cell annotations denoting malignant B cells, healthy B cells, and myeloid cells. Only cells annotated as malignant B cells were used for the analysis. In total, 8,235 cells were used for subsequent analysis.

Seurat (v4.3.0; ref. 69) was used for the analysis of the single-cell datasets. All functions were run with default parameters unless specified otherwise. Low-quality cells, defined by <200 genes per cell and >10% mitochondrial genes, were excluded from the analysis. Genes expressed in less than 3 cells were excluded from the analysis. The two datasets were integrated using the Seurat Integration protocol for data normalized with the “sctransform” method (RRID: SCR_022146; ref. 70); https://satijalab.org/seurat/articles/integration_introduction.html#performing-integration-on-datasets-normalized-with-sctransform-1. The data were integrated with each study and treated as a batch. Default parameters were used with 2,000 genes being used for the SelectIntegrationFeatures() function. Following this, based on the count data, each cell was assigned an expression status with double expressors being defined as below and the rest assigned as others.




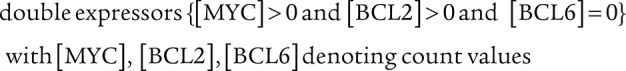




Differential gene-expression analysis was conducted using the FindMarkers() function in Seurat with ident.1 being M+2+6− cells and ident.2 being others. Nonparametric Wilcoxon rank sum test was used for the FindMarkers function. Next, functional enrichment was conducted using the gProfiler2 (v0.2.1; RRID: SCR_018190; ref. 71) package utilizing the WikiPathways (RRID:SCR_002134; ref. 72) database as source via the gost() function. FDR was the correction method used for multiple testing and all enriched pathways survived an FDR of 5%. Upregulated genes (defined by avg_log2FC >0 and *P* < 0.05) from the differential expression analysis were interrogated in gprofiler2.

### Software and Statistical Analysis

Graphical representations of data were generated in either GraphPad Prism 9 (RRID: SCR_000306) or R (RRID: SCR_001905). All relevant statistical tests were performed in GraphPad Prism 9 unless indicated otherwise. For Supplementary Table S3, Mann–Whitney and Kruskal–Wallis tests were performed using the R “stats” package; pooling of *P* values was performed using the R “poolr” package.

### Supplementary Methods

Additional details on IHC, spatial analysis, and unsupervised clustering can be found in the Supplementary Methods section within the Supplementary Appendix.

### Data Sharing Statement

- RNA-seq data generated from GC B cells overexpressing either MYC and BCL2 or MYC, BCL2, and BCL6 are deposited to GEO (RRID: SCR_005012; ref. 62) under the accession number: GSE203446.
- scRNA-seq data of GC primary B cells transduced either with BCL2 and MYC (MYC-transduced) or BCL2 and BCL6 (BCL6-transduced), or untransduced, have been made available as a Shiny app (68): https://bioinf.stemcells.cam.ac.uk/shiny/hodson/MYC-BCL2-BCL6_project.
- Source code for custom pipelines has been deposited to GitHub at the link: https://github.com/MichalMarekHoppe/Patterns-of-oncogene-co-expression-at-single-cell-resolution-influence-survival-in-lymphoma, and includes:
  ∘ “Mapping of MYC, BCL2, BCL6 mRNA DLBCL cohort expression data”
  ∘ “Pair correlation function clustering”
  ∘ “Spatial analysis – calculating delta between cellular phenotypes”
  ∘ “DLBCL scRNA-seq re-analysis”

- Publicly available gene-expression data for Sha and colleagues (17), GSE117556; McCord and colleagues (63), GSE125966; Visco and colleagues (18), GSE31312; Lenz and colleagues (19), GSE10846; Dubois and colleagues (20), GSE87371; Barrans and colleagues (21), GSE32918; and Chapuy and colleagues (22), GSE98588; can be found on GEO under the indicated accession numbers.
- Restricted access gene-expression data can be found at the following repositories under the respective accession numbers:
  ∘ Reddy and colleagues (23); The EGA (https://ega-archive.org/), Study ID: EGAS00001002606
  ∘ Schmitz and colleagues (24); NIH database of Genotypes and Phenotypes (dbGaP; RRID:SCR_002709), accession number: phs001444.v2.p1

- GEP-derived COO scores and LymphGen genetic subtype classifications for the BCA cohort and for Schmitz and colleagues were obtained from (49) and (24), respectively.
- Publicly available scRNA-seq data for Steen and colleagues (33) can be accessed through GEO under the accession number GSE182434; for Roider and colleagues (34) can be accessed through the heiDATA database (https://heidata.uni-heidelberg.de) from https://doi.org/10.11588/data/VRJUNV.

## Supplementary Material

Supplementary tables Supplementary table 1. Per-patient mfIHC MYC, BCL2 and BCL6 single oncogene and subpopulation scores for normal tonsil tissue and reactive lymph node tissue. Supplementary table 2. Per-patient mfIHC MYC, BCL2 and BCL6 single oncogene and subpopulation scores for DLBCL tissue (NUH, CMMC, SGH, MDA, BCA and UP). Supplementary table 6. Inferred percentage extents of MYC, BCL2, BCL6 and sub-population metrics in GEP cohorts. Supplementary table 11. Correlation of M+2+6- metric with gene expression in GEP cohorts. Supplementary table 12. Differential gene expression analysis of primary germinal center (GC) B-cells with M+2+ and M+2+6+ overexpression. Supplementary table 13. Differentially expressed genes between M+2+6- and all other malignant cells in scRNA-seq samples of DLBCL. Dichotomized non-parametric comparison, Wilcoxon rank sum test. Supplementary table 14. Analysis of positive enrichment of Wikipathways terms between M+2+6- and all other malignant cells in scRNA-seq samples of DLBCL by gprofiler2.

Supplementary appendix Supplementary methods. Supplementary Figure 1. Phenotyping of B-cells in non-malignant tissues. A, Quantitation of marker positivity across ten tonsil and two reactive lymph node samples (rLN). Analysis is spatially resolved between the GC and extra-GC zones. B, Spatial map of cellular coordinates based on cell segmentation of images in Figure 1B. Marker-positivity is indicated, and a total proportion of positive and negative cells is depicted as a pie chart. These maps were used to derive sub-population phenotypes depicted in Figure 1C. Scale bar is 100μm. C, Proliferation analysis (i.e., Ki67-positivity) among sub-populations in five tonsil samples. Median with interquartile range, whiskers denote 10th and 90th percentile. Supplementary figure 2. Example pseudo-colored mfIHC images for MYC, BCL2, BCL6 cases in DLBCL. Images of a range of mean fluorescent intensities are shown with equal scaling for reference. Supplementary figure 3. Global distribution of MYC, BCL2 and BCL6 sub-populations within DLBCL cohorts. Heat-maps displaying the percentage extent of individual markers and each sub-population within the DLBCL NUH, CMMC, SGH and MDA cohorts. Hierarchical k-means clustering of patients according to sub-population extent is applied. Positivity shading for single markers ranges between 0-100% positivity, whereas shading for sub-populations reflects 0-50% positivity and remains fully saturated until 100%. IPI Risk Group - International Prognostic Index Risk Group, FISH - fluorescence in situ hybridization. Supplementary figure 4. Intra-tumor heterogeneity of sub-populations. A, Correlation of sub-population extent quantification between two biopsies of the same patient for which at least two tissue microarray (TMA) biopsies are available. Correlation is shown separately for lymph node and extranodal biopsies. Spearman rho is indicated for each correlation. Axes are in exponential and equivalent in all panels. B, Sub-population percentage extent quantification across multiple TMA cores (columns) of the same patient (rows). Pie charts are ordered according to decreasing cell numbers evaluated per core. All patients from the NUH cohort with at least five cores are evaluated. A heterogenous cluster is highlighted by the red box. Supplementary figure 5. Spatial heterogeneity of sub-population interactions. A, Conceptual schematic of pair correlation function (PCF) plots depicting a clustered distribution (left, green) and a random distribution (right, grey). Representative counterpart spatial maps are above each plot. B, PCF analysis for sub-populations to investigate spatial clustering (top). Mean results for two independent cohorts (shading is cohort standard deviation). An example tissue microarray core is shown as physical distance reference for spatial analyses (bottom left). Absolute number of neighboring cells expected within a given radius (data from 3500 randomly selected cells across all images, mean with standard deviation) (bottom right). C, Actual spatial map of sub-populations of an example DLBCL case (top). Extent of all sub-populations within the sample is shown on the left. Simulated, hypothetical random distribution of cells for the same case (middle). PCF analysis for the shown sample and its matched simulated random distribution (bottom). Scale bars in B and C are 100µm. D, Mean deviations from expected neighbor abundance (Δ%) summarizing cell-cell interactions between sub-populations for the sample shown in (C). E, Sub-population interaction matrices from spatially distinct biopsies (cores in tissue microarray) for example DLBCL patients. Biopsies of stable, spatially homogenous, sub-population interaction profiles are grouped (top), whereas biopsies of a differing, heterogenous, interaction profile are grouped separately (bottom). Supplementary figure 6. Global deviations from expected spatial neighbor abundance (Δ%). Hierarchical clustering (minimum variance method) of measured Δ% for all cases in the SGH and MDA cohorts. Extents of sub-populations are indicated for reference (top). For the MDA cohort, multiple biopsies (n = 1-3) from the same patient were included in the analysis to determine spatial interaction similarity across spatially distinct regions (bottom). Supplementary figure 7. Correlation of predicted MYC, BCL2 and BCL6 sub-population percentage extent based on single oncogene positivity and observed percentage extent in DLBCL cohorts. Spearman rho, axes are equivalent in all panels. Supplementary figure 8. Variance of M+2+6- percentage extent in the context of positivity calling across a 15% cut-off. A, M+2+6- scoring variance across multiple pathological imaging fields. All whole-tissue DLBCL sections from University of Palermo (UP), and samples from the NUH TMA with at least four fields scored per patient and a mean M+2+6- score above 5% are shown. Mean with SD. Ordinates between 50-100% are compressed for clarity. Dashed line denotes M+2+6- 15% positivity. B, Stability of M+2+6- case positivity calling across scoring increasing number of imaging fields. All cases from panel A with at least five fields scored in this study are shown. Only one case is called M+2+6- Low (<15%) at the first image scored, and subsequently called M+2+6- High (≥15%) after two or more fields scored. Supplementary figure 9. Mapping of mRNA expression data into percentage extent data. A, Cumulative histogram of MYC, BCL2 and BCL6 protein percentage extent positivity in DLBCL cohorts (data transformed from Figure 4A) (top). B, Aggregated single oncogene cumulative distribution of MYC, BCL2 and BCL6 protein percentage extent positivity across all measured protein cohorts and its smoothed empirical cumulative distribution function (eCDF).C, Distribution of inferred single oncogene percentage extent in GEP cohorts. (see Supplementary table 6 for all values). Supplementary figure 10. Analysis of the GOYA clinical trial. A, Correlation of MYC mRNA with quantitative IHC score. Linear regression (left) and Wilcoxon rank sum test (right). B, Analysis as in (A) for BCL2. C, Kaplan-Meier curves for PFS and OS for patients stratified across the 15% M+2+6- metric (GEP-derived). Multivariate Cox proportional hazards model is available in Supplementary table 9. PFS - progression free survival, OS - overall survival. Supplementary figure 11. Proliferative advantage of cyclin D2 (CCND2) overexpressing B-cells. Representative FACS plots documenting to the expansion over time of the cyclin D2 positive GC B-cell population in cyclin D2 overexpressing GC B-cells (CCND2-Lyt2) and non-cyclin D2 overexpressing GC B-cells (Empty vector Lyt2). All GC B-cells co-overexpress BCL2, BCL6, MYC and GFP. Supplementary table 3. Non-parametric correlation of sub-population percentage extent with clinicopathological features. Supplementary table 4. Pooled univariate analysis for MYC, BCL2 and BCL6 single oncogene and sub-populations percentage extents as a continuous variable at 5% increments as predictors for overall survival (OS) in mfIHC cohorts of DLBCL (Cox proportional hazards model). Supplementary table 5. Univariate analysis of clinicopathological features as a predictor of overall survival (OS) after first-line R-CHOP treatment in the NUH, SGH and MDA cohorts of DLBCL (Cox proportional hazards model). Supplementary table 7. Pooled univariate analysis for sub-population metrics as a continuous variable at 5% increments as predictors for overall survival (OS) in GEP DLBCL cohorts (Cox proportional hazards model). Supplementary table 8. Multivariate analysis of continuous M+2+6- metric at 5% increments as a predictor of overall survival (OS) in cohorts with gene-expression data (Cox proportional hazards model). Supplementary table 9. Univariate and multivariate analysis of continuous M+2+6- metric as a continuous variable at 5% increments as predictor of progression-free survival (PFS) and overall survival (OS) in the GOYA trial cohort (Cox proportional hazards model). Supplementary table 10. Multivariate analysis of M+2+6- metric dichotomized at 15% as a predictor of overall survival (OS) in cohorts with gene-expression data (Cox proportional hazards model). Supplementary table 15. Clinicopathologic characteristics of DLBCL patients evaluated by multiplexed fluorescent immunohistochemistry (mfIHC) in this study. Supplementary table 16. Manual multiplexed fluorescent immunohistochemistry (mfIHC) staining protocol performed on the NUH and CMMC cohort TMA. Supplementary table 17. Automated multiplexed fluorescent immunohistochemistry (mfIHC) staining protocol performed on the SGH, MDA and BCA cohort TMA.
