# Supplementary material for: Patterns of Oncogene Coexpression at Single-Cell Resolution Influence Survival in Lymphoma
Source: Cancer Discov. 2023 Apr 18;13(5):1144–63. doi: 10.1158/2159-8290.CD-22-0998 (PMC10157367; doi:10.1158/2159-8290.CD-22-0998)
Supplement: Supplementary appendix — Supplementary methods. Supplementary Figure 1. Phenotyping of B-cells in non-malignant tissues. A, Quantitation of marker positivity across ten tonsil and two reactive lymph node samples (rLN). Analysis is spatially resolved between the GC and extra-GC zones. B, Spatial map of cellular coordinates based on cell segmentation of images in Figure 1B. Marker-positivity is indicated, and a total proportion of positive and negative cells is depicted as a pie chart. These maps were used to derive sub-population phenotypes depicted in Figure 1C. Scale bar is 100μm. C, Proliferation analysis (i.e., Ki67-positivity) among sub-populations in five tonsil samples. Median with interquartile range, whiskers denote 10th and 90th percentile. Supplementary figure 2. Example pseudo-colored mfIHC images for MYC, BCL2, BCL6 cases in DLBCL. Images of a range of mean fluorescent intensities are shown with equal scaling for reference. Supplementary figure 3. Global distribution of MYC, BCL2 and BCL6 sub-populations within DLBCL cohorts. Heat-maps displaying the percentage extent of individual markers and each sub-population within the DLBCL NUH, CMMC, SGH and MDA cohorts. Hierarchical k-means clustering of patients according to sub-population extent is applied. Positivity shading for single markers ranges between 0-100% positivity, whereas shading for sub-populations reflects 0-50% positivity and remains fully saturated until 100%. IPI Risk Group - International Prognostic Index Risk Group, FISH - fluorescence in situ hybridization. Supplementary figure 4. Intra-tumor heterogeneity of sub-populations. A, Correlation of sub-population extent quantification between two biopsies of the same patient for which at least two tissue microarray (TMA) biopsies are available. Correlation is shown separately for lymph node and extranodal biopsies. Spearman rho is indicated for each correlation. Axes are in exponential and equivalent in all panels. B, Sub-population percentage extent quantification acr [file cd-22-0998_supplementary_appendix_suppsm1.docx]

**Supplementary appendix: Patterns of oncogene co-expression at single cell resolution influence survival in lymphoma**

Contents

[Supplementary methods 1](#_Toc126832203)

[Semi-quantitative immunohistochemistry 1](#_Toc126832204)

[Spatial analysis 1](#_Toc126832205)

[Unsupervised clustering 3](#_Toc126832206)

[Supplementary figures 4](#_Toc126832207)

[Supplementary tables 18](#_Toc126832208)

[References 32](#_Toc126832209)

# Supplementary methods

## Semi-quantitative immunohistochemistry

TMA preparation and staining for MYC, BCL2 and BCL6 for the BCA cohort are described in ref. (1) and ref. (2). Percentage extent of MYC, BCL2 and BCL6 staining was determined independently by two pathologists. Thresholds for overall tumor positivity for a given oncogene were: 40% for MYC, 50% for BCL2 and 30% for BCL6. When there was a discrepancy in scoring between the two pathologists to the extent that scores were found to be on opposing sides of a positivity threshold, a consensus score was obtained from a third pathologist.

## Spatial analysis

The difference in clustering of different oncogene (MYC, BCL2 and BCL6) sub-populations in DLBCL were evaluated for images from the SGH (67 images) and the MDA (87 images) cohorts. We annotated the images and used the point pattern processes to estimate the clustering. In order to identify clustering in different sub-populations we used the Pair-Correlation Function (PCF) (3,4).

The PCF is the observed number of pairs of cells that are about *r* units apart, divided by the expected number that would be obtained if the cells were completely random. Mathematically, it is given by,

$$g\left( r \right)= \frac{K'(r)}{2\pi r}$$

where $K(r)$ is the Ripley’s *K*-function that provides an estimate for the expected number of cells within radius *r* of any given cell. This measure can determine if the cells are inhibiting, random or clustering.

First, raw images were generated with a complete tissue mask in inForm2.4.8 (RRID: SCR_019155). We annotated these raw images in QuPath (RRID: SCR_018257) by overlaying the cell coordinates using an open source package (opencv2). The annotations were stored as geojson files from which the spatial point pattern objects were generated for each image. For the point pattern objects generated for each cohort, each of the sub-populations were extracted and the clustering was estimated using the PCF function in spatstat (5), a comprehensive spatial statistics package. Following this, a distribution of the PCF values for all values of *r* for a specific sub-population was plotted. The mode around 1 will correspond to the random subset of the cells, while the modes less than and greater than 1 denotes inhibited and clustering subsets, respectively. We performed this analysis for each sub-population for both SGH and MDA cohorts.

To investigate spatial relationships between different sub-populations, we investigated the sub-population identity of *k*=20 nearest neighbors for each cell (using beforementioned spatial coordinates and a custom base R script available at GitHub (RRID: SCR_002630): <https://github.com/MichalMarekHoppe/Patterns-of-oncogene-co-expression-at-single-cell-resolution-influence-survival-in-lymphoma.git>) and compared the percentage extent of such local neighborhood samples to the overall observed sub-populations extent for a given image. The absolute difference between observed local and global sub-population percentage for each cell we term Δ% and a mean value for all cells of the same sub-population in an image we define as a measure of sub-population to sub-population integration or separation.

## Unsupervised clustering

Unsupervised Euclidean Ward clustering was performed on percentage extents within patient cohorts (see Supplementary figure 3) and on measured Δ% for all cases in the SGH and MDA cohorts (see Supplementary figure 6) using the R packages ‘cluster’ (RRID: SCR_013505) and ‘factoextra’ (RRID: SCR_016692).

# Supplementary figures


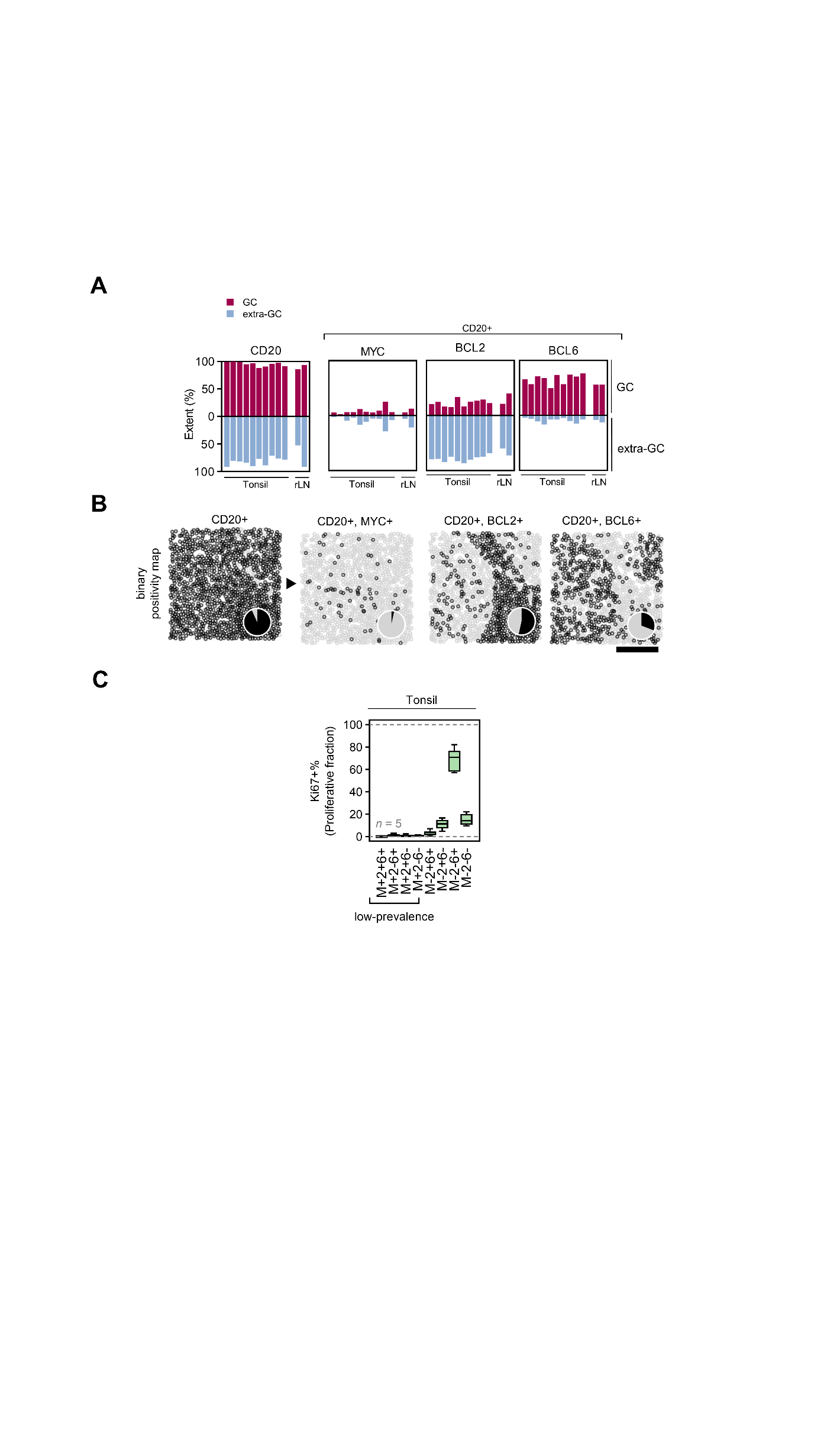


**Supplementary Figure 1. Phenotyping of B-cells in non-malignant tissues**. A, Quantitation of marker positivity across ten tonsil and two reactive lymph node samples (rLN). Analysis is spatially resolved between the GC and extra-GC zones. B, Spatial map of cellular coordinates based on cell segmentation of images in Figure 1B. Marker-positivity is indicated, and a total proportion of positive and negative cells is depicted as a pie chart. These maps were used to derive sub-population phenotypes depicted in Figure 1C. Scale bar is 100μm. C, Proliferation analysis (i.e., Ki67-positivity) among sub-populations in five tonsil samples. Median with interquartile range, whiskers denote 10th and 90th percentile.

**
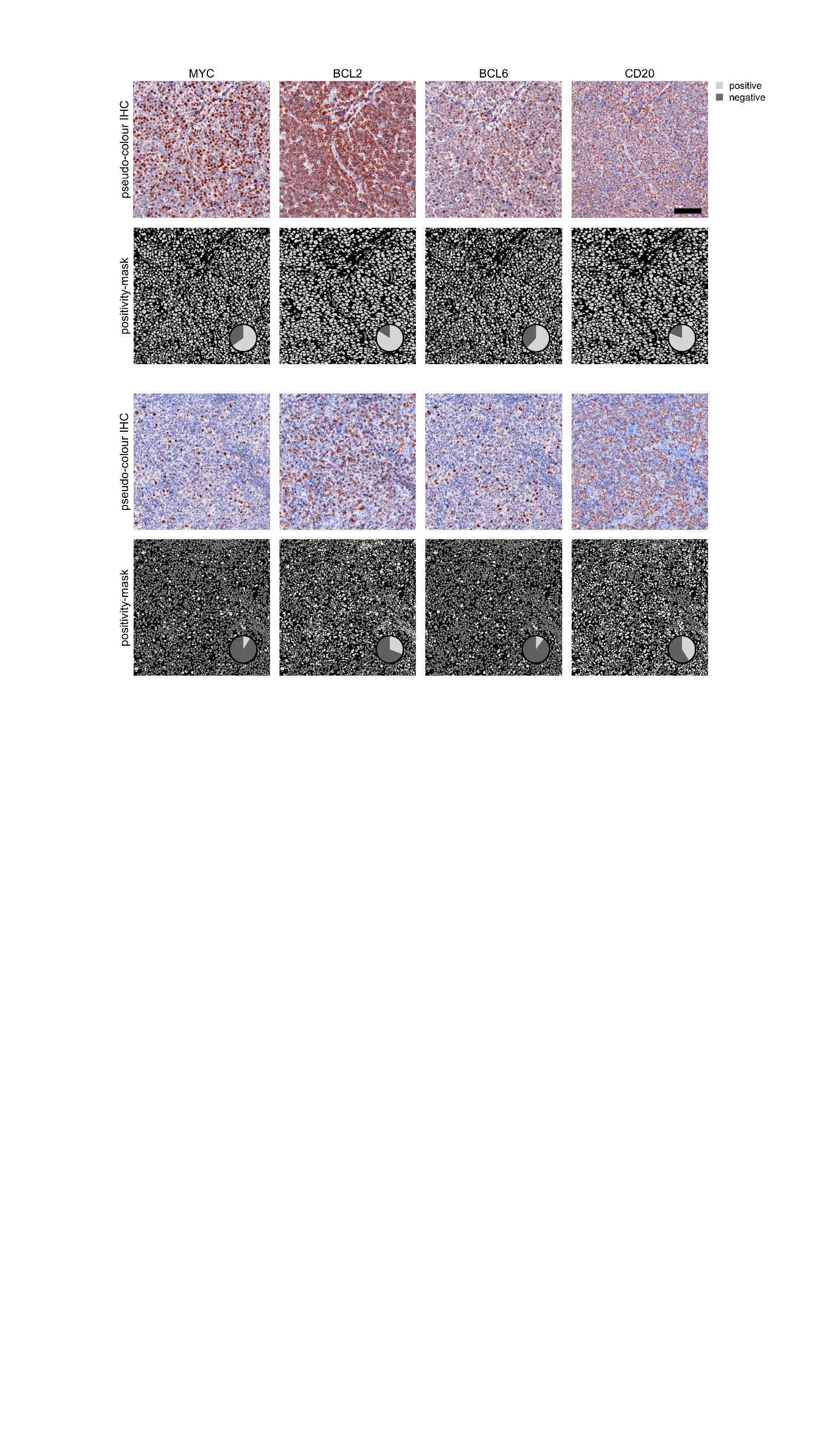
**

**Supplementary figure 2. Example pseudo-colored mfIHC images for MYC, BCL2, BCL6 cases in DLBCL.** Images of a range of mean fluorescent intensities are shown with equal scaling for reference.


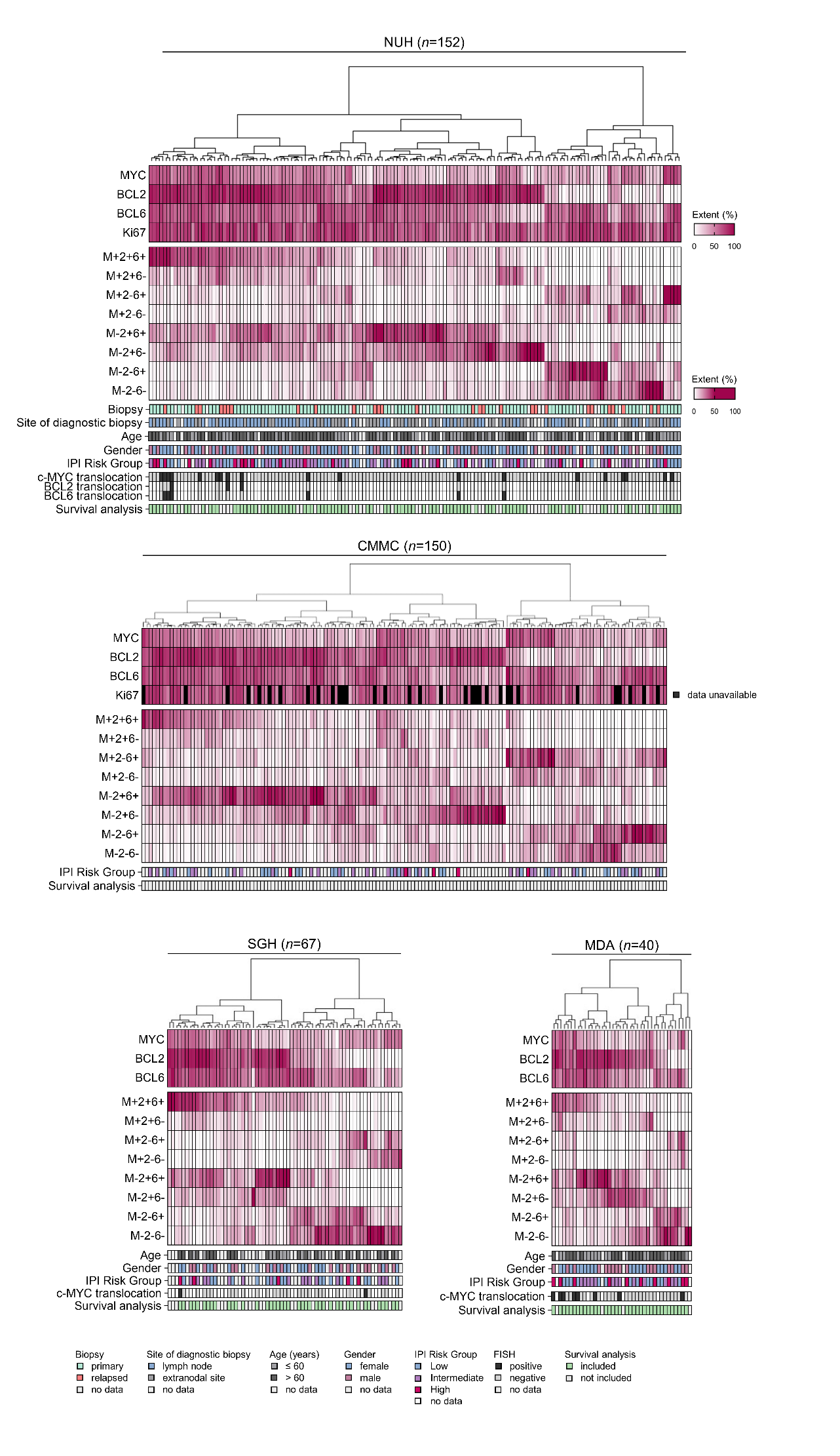


**Supplementary figure 3. Global distribution of MYC, BCL2 and BCL6 sub-populations within DLBCL cohorts.** Heat-maps displaying the percentage extent of individual markers and each sub-population within the DLBCL NUH, CMMC, SGH and MDA cohorts. Hierarchical k-means clustering of patients according to sub-population extent is applied. Positivity shading for single markers ranges between 0-100% positivity, whereas shading for sub-populations reflects 0-50% positivity and remains fully saturated until 100%. IPI Risk Group - International Prognostic Index Risk Group, FISH - fluorescence in situ hybridization.


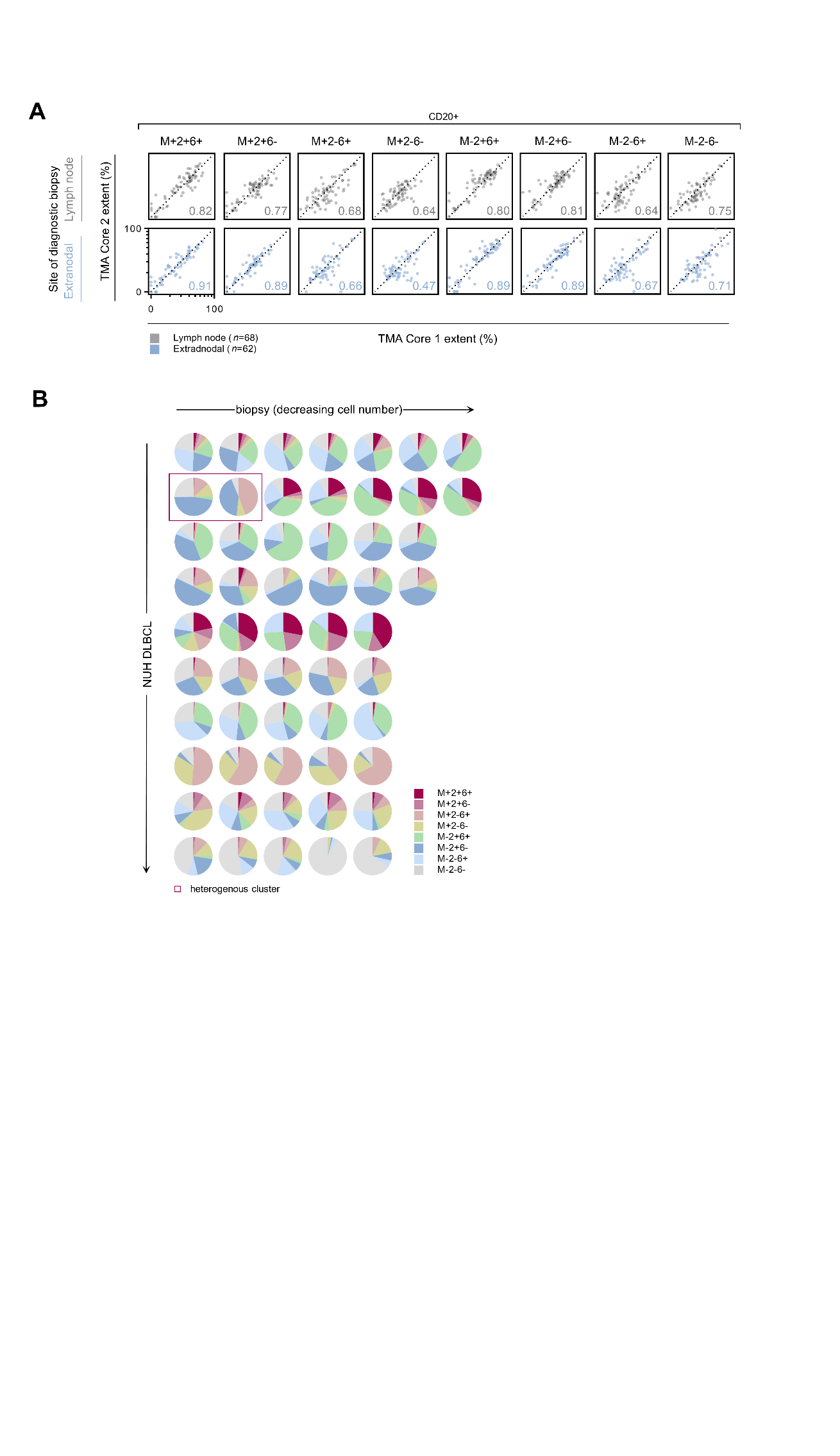


**Supplementary figure 4. Intra-tumor heterogeneity of sub-populations.** A, Correlation of sub-population extent quantification between two biopsies of the same patient for which at least two tissue microarray (TMA) biopsies are available. Correlation is shown separately for lymph node and extranodal biopsies. Spearman rho is indicated for each correlation. Axes are in exponential and equivalent in all panels. B, Sub-population percentage extent quantification across multiple TMA cores (columns) of the same patient (rows). Pie charts are ordered according to decreasing cell numbers evaluated per core. All patients from the NUH cohort with at least five cores are evaluated. A heterogenous cluster is highlighted by the red box.


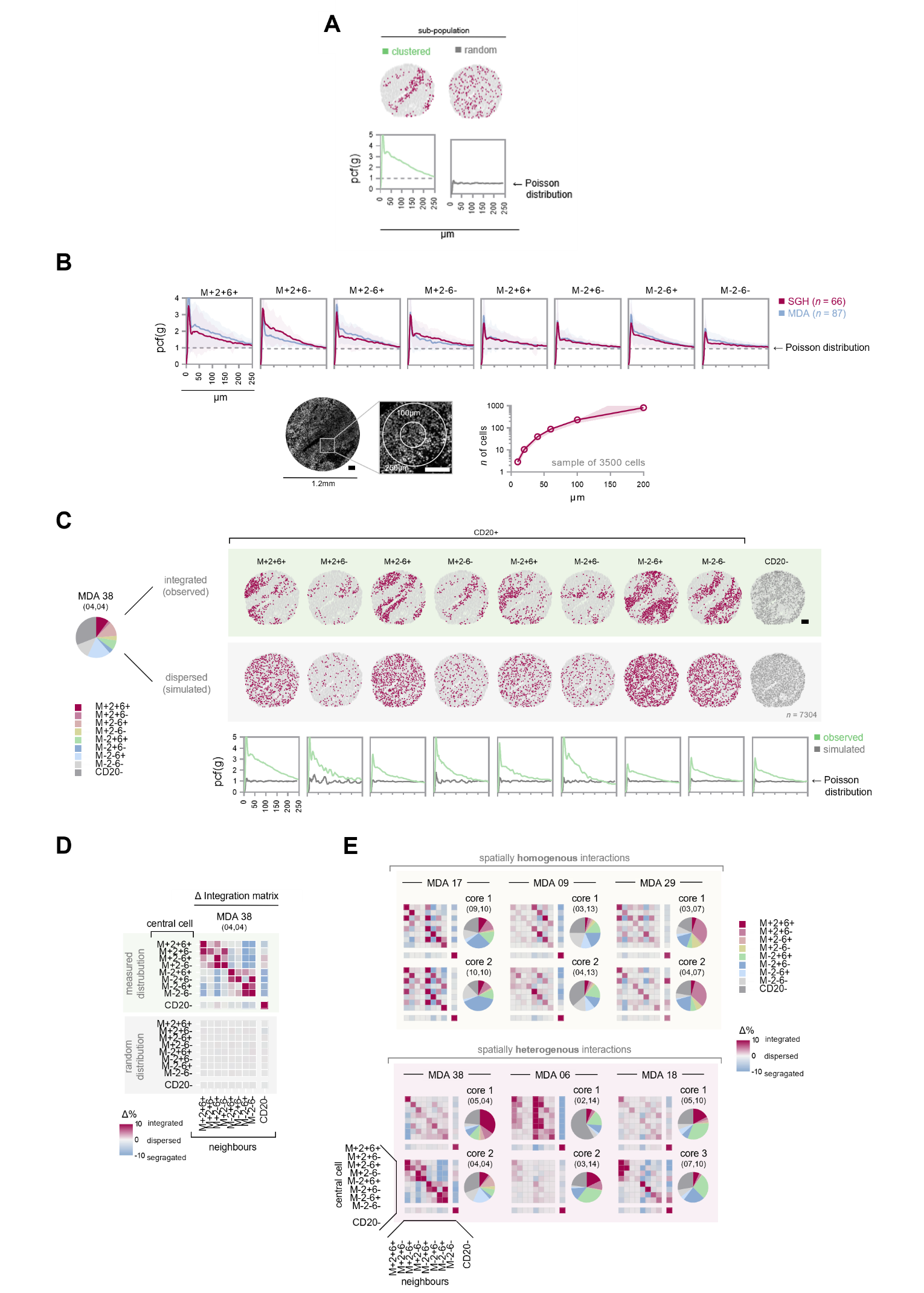


**Supplementary figure 5. Spatial heterogeneity of sub-population interactions.** A, Conceptual schematic of pair correlation function (PCF) plots depicting a clustered distribution (*left, green*) and a random distribution (*right, grey*). Representative counterpart spatial maps are above each plot. B, PCF analysis for sub-populations to investigate spatial clustering (*top*)*.* Mean results for two independent cohorts (shading is cohort standard deviation). An example tissue microarray core is shown as physical distance reference for spatial analyses (*bottom left*). Absolute number of neighboring cells expected within a given radius (data from 3500 randomly selected cells across all images, mean with standard deviation) (*bottom right*). C, Actual spatial map of sub-populations of an example DLBCL case (*top*). Extent of all sub-populations within the sample is shown on the left. Simulated, hypothetical random distribution of cells for the same case (*middle*). PCF analysis for the shown sample and its matched simulated random distribution (*bottom*). Scale bars in B and C are 100µm. D, Mean deviations from expected neighbor abundance (Δ%) summarizing cell-cell interactions between sub-populations for the sample shown in (C). E, Sub-population interaction matrices from spatially distinct biopsies (cores in tissue microarray) for example DLBCL patients. Biopsies of stable, spatially homogenous, sub-population interaction profiles are grouped (*top*), whereas biopsies of a differing, heterogenous, interaction profile are grouped separately (*bottom*).


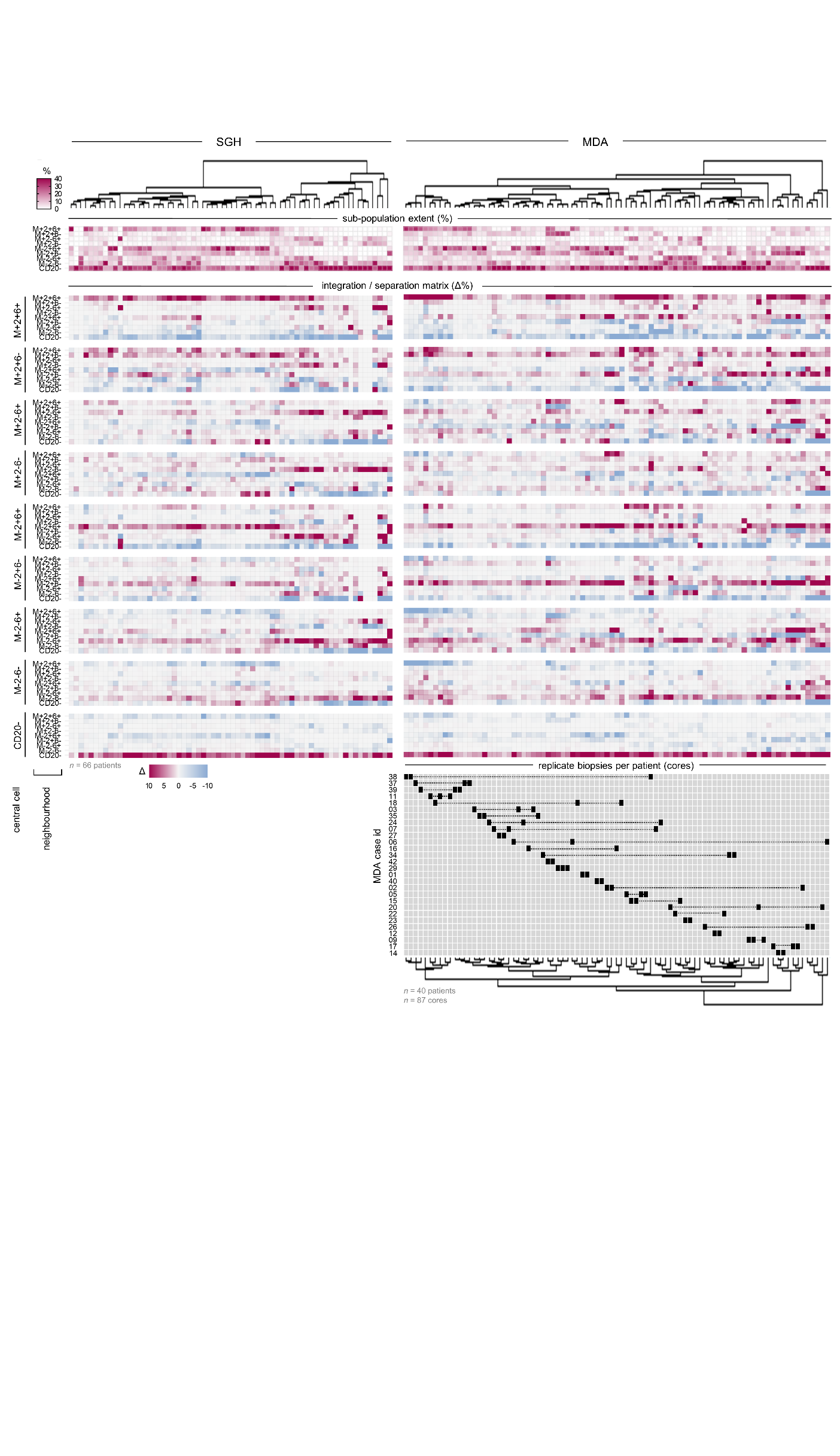


**Supplementary figure 6. Global deviations from expected spatial neighbor abundance (Δ%).** Hierarchical clustering (minimum variance method) of measured Δ% for all cases in the SGH and MDA cohorts. Extents of sub-populations are indicated for reference (*top*)*.* For the MDA cohort, multiple biopsies (*n =* 1-3) from the same patient were included in the analysis to determine spatial interaction similarity across spatially distinct regions (*bottom*)*.*


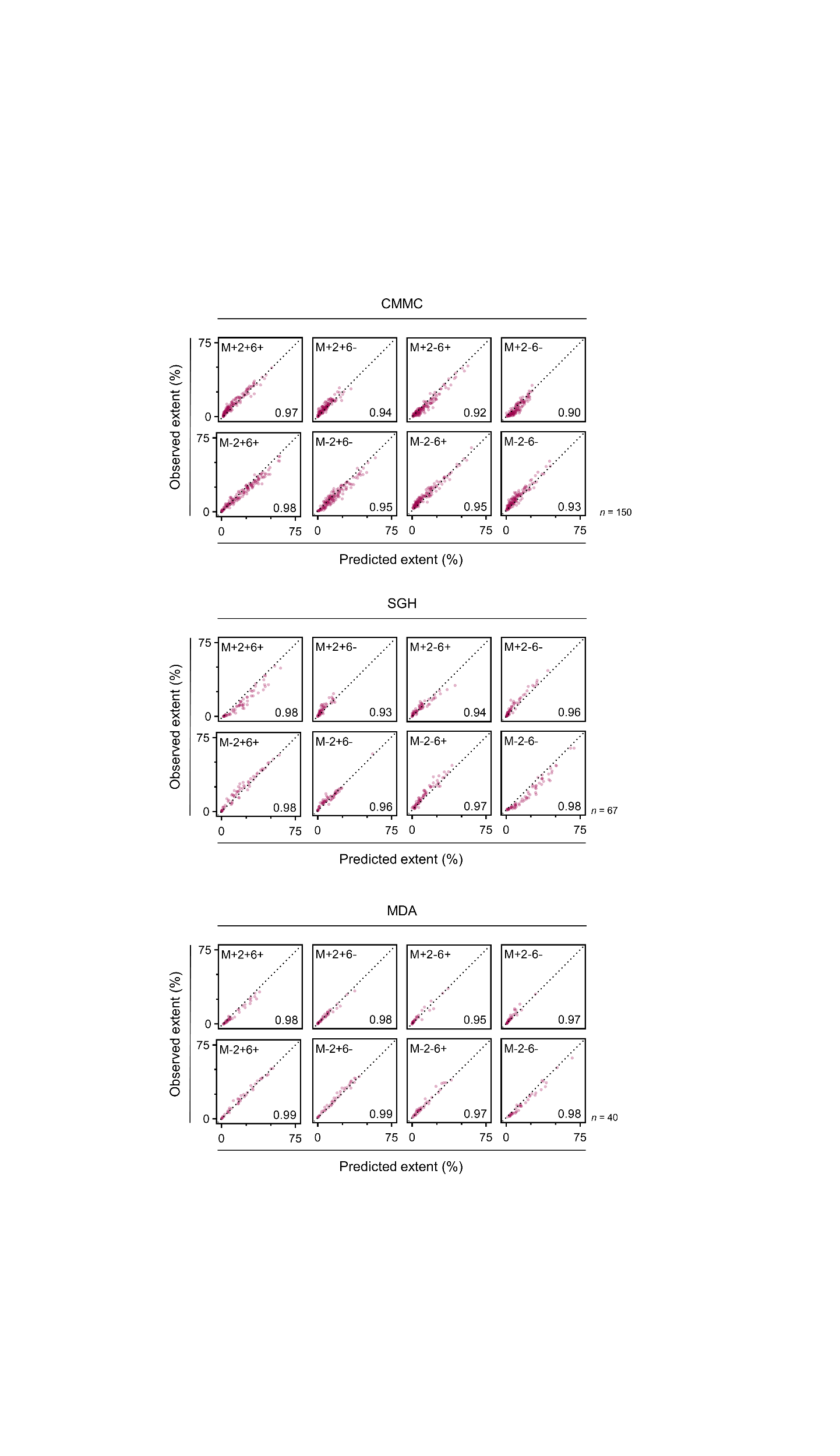


**Supplementary figure 7. Correlation of predicted MYC, BCL2 and BCL6 sub-population percentage extent based on single oncogene positivity and observed percentage extent in DLBCL cohorts.** Spearman rho, axes are equivalent in all panels.


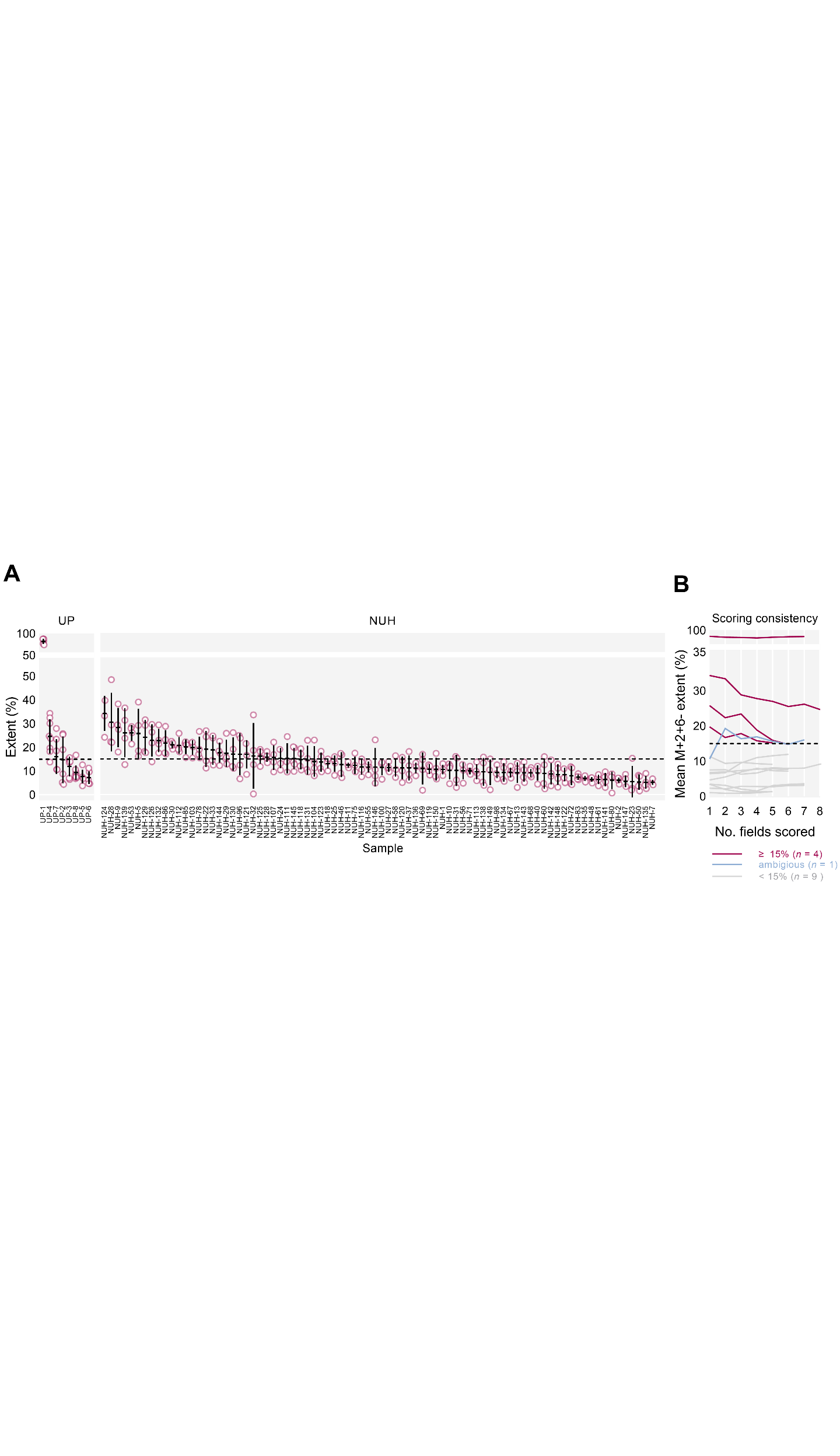


**Supplementary figure 8. Variance of M+2+6- percentage extent in the context of positivity calling across a 15% cut-off.** A, M+2+6- scoring variance across multiple pathological imaging fields. All whole-tissue DLBCL sections from University of Palermo (UP), and samples from the NUH TMA with at least four fields scored per patient and a mean M+2+6- score above 5% are shown. Mean with SD. Ordinates between 50-100% are compressed for clarity. Dashed line denotes M+2+6- 15% positivity. B, Stability of M+2+6- case positivity calling across scoring increasing number of imaging fields. All cases from panel A with at least five fields scored in this study are shown. Only one case is called M+2+6- Low (<15%) at the first image scored, and subsequently called M+2+6- High (≥15%) after two or more fields scored.


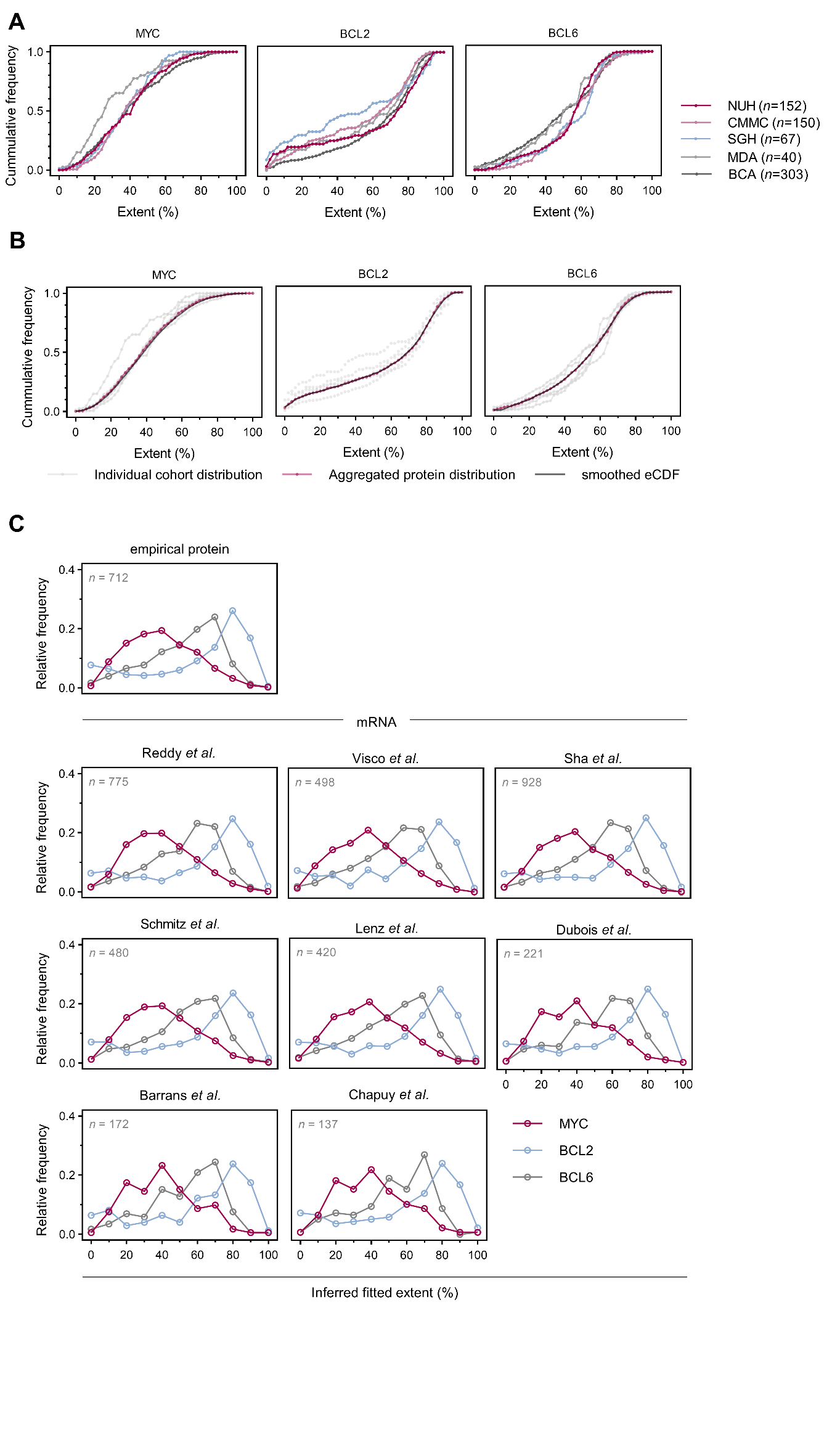


**Supplementary figure 9. Mapping of mRNA expression data into percentage extent data**. A, Cumulative histogram of MYC, BCL2 and BCL6 protein percentage extent positivity in DLBCL cohorts (data transformed from Figure 4A) (*top*). B, Aggregated single oncogene cumulative distribution of MYC, BCL2 and BCL6 protein percentage extent positivity across all measured protein cohorts and its smoothed empirical cumulative distribution function (eCDF).C, Distribution of inferred single oncogene percentage extent in GEP cohorts. (see Supplementary table 6 for all values).


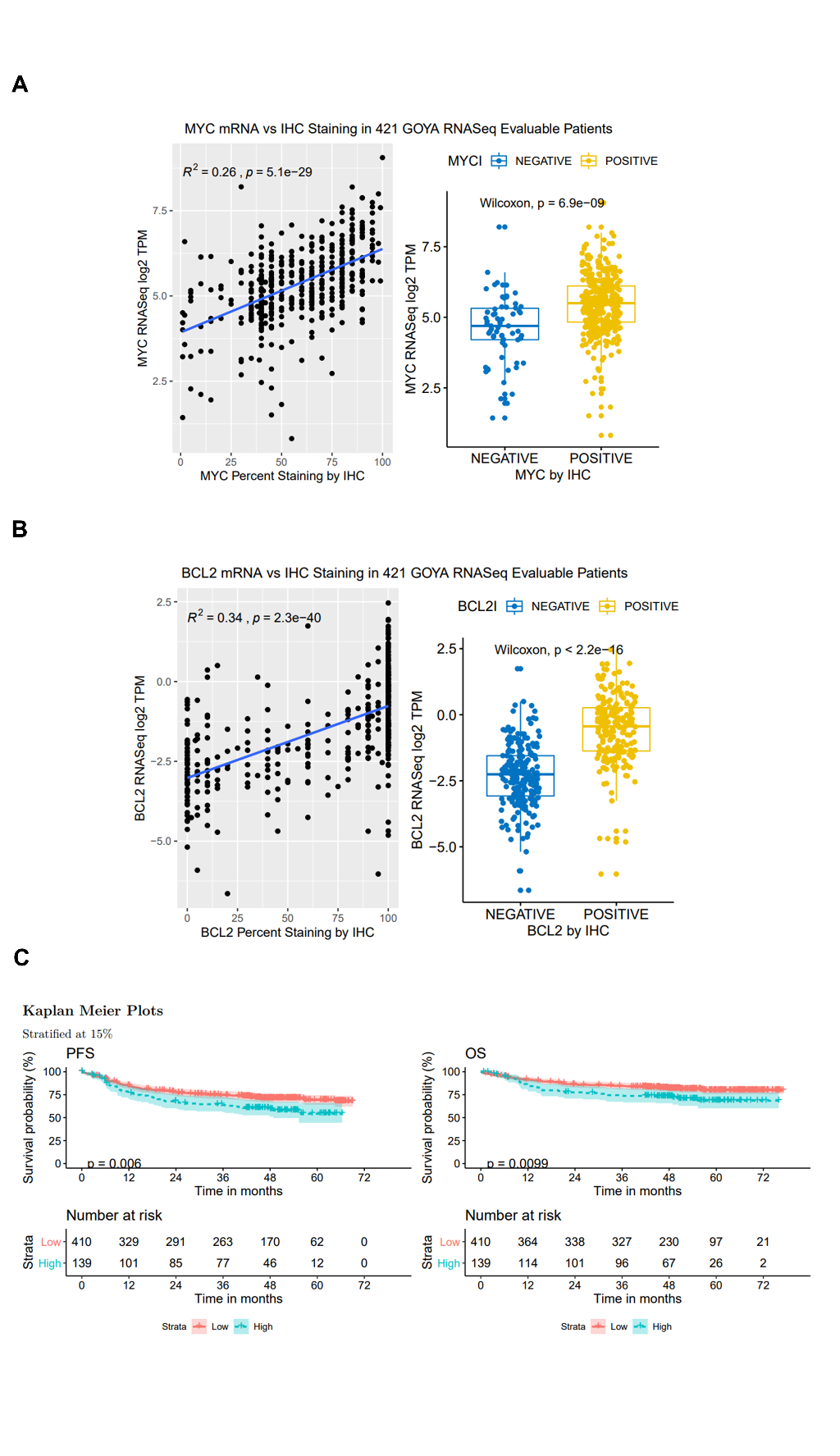


**Supplementary figure 10. Analysis of the GOYA clinical trial.** A, Correlation of MYC mRNA with quantitative IHC score. Linear regression (*left*) and Wilcoxon rank sum test (*right*). B, Analysis as in (A) for BCL2. C, Kaplan-Meier curves for PFS and OS for patients stratified across the 15% M+2+6- metric (GEP-derived). Multivariate Cox proportional hazards model is available in Supplementary table 9. PFS - progression free survival, OS - overall survival.


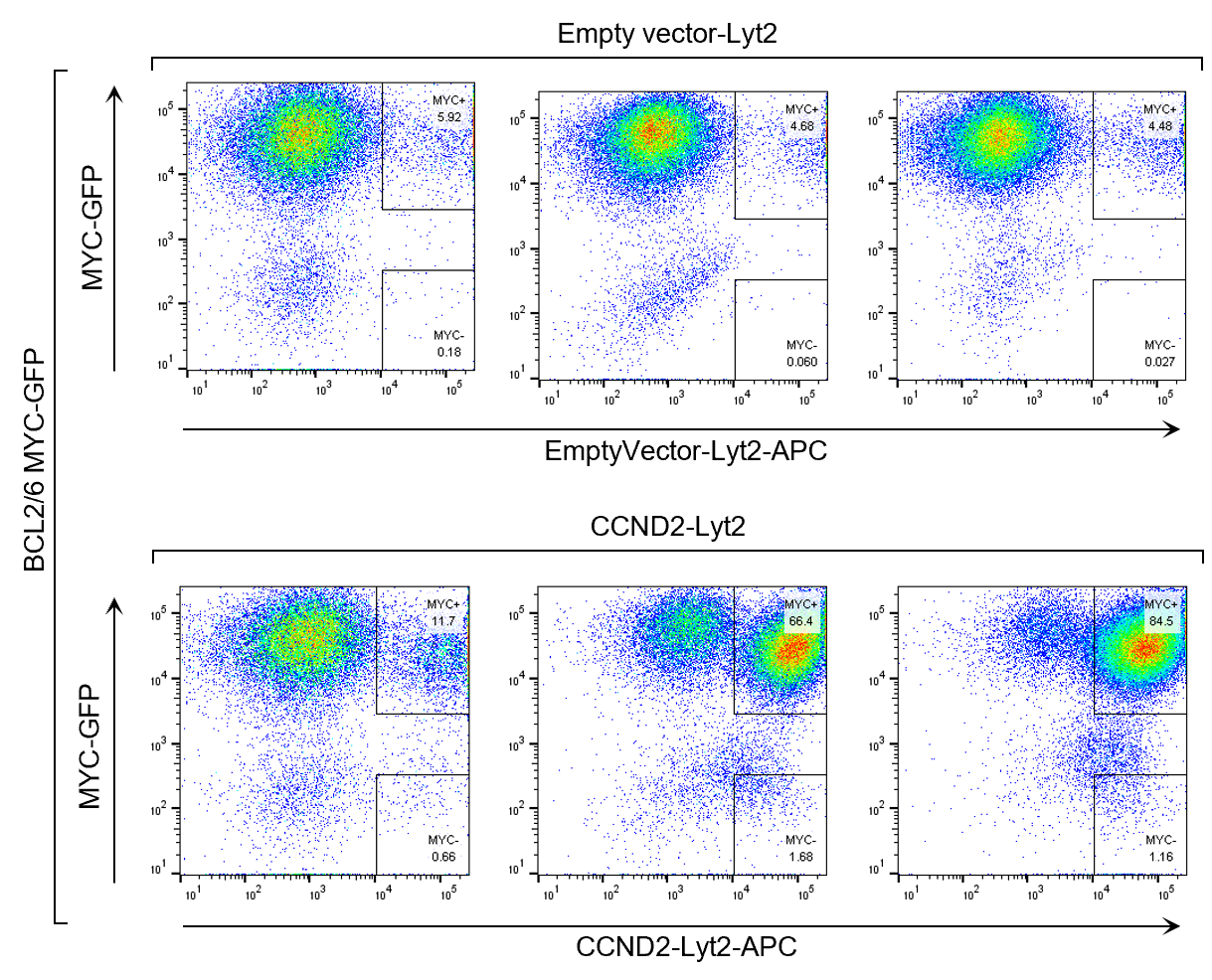


**Supplementary figure 11. Proliferative advantage of cyclin D2 (*CCND2*) overexpressing B-cells.** Representative FACS plots documenting to the expansion over time of the cyclin D2 positive GC B-cell population in cyclin D2 overexpressing GC B-cells (CCND2-Lyt2) and non-cyclin D2 overexpressing GC B-cells (Empty vector Lyt2). All GC B-cells co-overexpress BCL2, BCL6, MYC and GFP.

# Supplementary tables

Supplementary table 1 – in a separate Excel file

**Supplementary table 1**. Per-patient mfIHC MYC, BCL2 and BCL6 single oncogene and subpopulation scores for normal tonsil tissue and reactive lymph node tissue.

Supplementary table 2 – in a separate Excel file

**Supplementary table 2**. Per-patient mfIHC MYC, BCL2 and BCL6 single oncogene and subpopulation scores for DLBCL tissue (NUH, CMMC, SGH, MDA, BCA and UP).

| Supplementary table 3. Non-parametric correlation of sub-population percentage extent with clinicopathological features. | | | | | | | | | | | | | | | | | | | | | | |
| --- | --- | --- | --- | --- | --- | --- | --- | --- | --- | --- | --- | --- | --- | --- | --- | --- | --- | --- | --- | --- | --- | --- |
|  | | | | | | | | | | | | | | | | | | | | | | |
|  | NUH | | | | | CMMC | | | | | SGH | | | | | MDA | | | | |  |  |
|  |  | | | | |  | | | | |  | | | | |  | | | | |  |  |
|  | Age | | | | | | | | | | | | | | | | | | | | | |
|  | ≤ 60  (*n*=42) | | > 60  (*n*=59) | |  | ≤ 60 | | > 60 | |  | ≤ 60  (*n*=20) | | > 60  (*n*=21) | |  | ≤ 60  (*n*=16) | | > 60  (*n*=20) | |  |  |  |
| Sub-population | median  (25^th^-75^th^ perc.) | | | | *p*-value* |  | |  | | *p*-value | median  (25^th^-75^th^ perc.) | | | | *p*-value* | median  (25^th^-75^th^ perc.) | | | | *p*-value* | pooled  *p*-value^#^ | adjusted  pooled  *p*-value^ |
| M+2+6+ | 9.7  (0.6-21.2) | | 16.8  (3.9-25.0) | | 0.085 | - | | - | | - | 10.6  (4.9-17.8) | | 20.2  (3.3-30.3) | | 0.122 | 10.7  (3.2-20.2) | | 6.2  (3.9-14.3) | | 0.765 | 0.139 | 1.000 |
| M+2+6- | 5.2  (1.2-9.5) | | 11.1  (4.8-15.3) | | 0.001 | - | | - | | - | 2.1  (1.0-4.1) | | 3.3  (2.1-6.2) | | 0.315 | 6.4  (2.3-10.4) | | 8.8  (2.7-10.9) | | 0.694 | 0.013 | 0.104 |
| M+2-6+ | 7.7  (4.4-13.5) | | 4.3  (2.4-9.5) | | 0.039 | - | | - | | - | 5.2  (1.7-11.0) | | 5.5  (1.4-11.3) | | 0.990 | 1.7  (1.4-4.0) | | 4.2  (1.9-8.3) | | 0.124 | 0.099 | 0.792 |
| M+2-6- | 5.3  (3.0-11.1) | | 4.3  (2.8-9.0) | | 0.570 | - | | - | | - | 3.1  (0.8-9.7) | | 3.0  (0.7-7.4) | | 0.990 | 1.7  (0.8-5.9) | | 4.1  (2.8-9.0) | | 0.039 | 0.266 | 1.000 |
| M-2+6+ | 19.0  (5.3-27.1) | | 22.0  (12.3-31.5) | | 0.105 | - | | - | | - | 16.6  (1.5-34.6) | | 15.2  (3.9-28.0) | | 0.928 | 30.8  (6.7-40.1) | | 17.1  (7.6-19.3) | | 0.168 | 0.222 | 1.000 |
| M-2+6- | 15.4  (5.8-24.1) | | 17.4  (9.8-25.9) | | 0.391 | - | | - | | - | 7.5  (1.8-18.7) | | 8.5  (4.9-15.4) | | 0.989 | 17.8  (12.4-24.5) | | 13.6  (4.3-28.2) | | 0.539 | 0.792 | 1.000 |
| M-2-6+ | 7.3  (4.4-17.6) | | 4.5  (2.9-10.9) | | 0.046 | - | | - | | - | 12.4  (5.9-21.6) | | 8.6  (4.4-14.3) | | 0.670 | 6.8  (4.2-12.6) | | 9.3  (6.8-27.1) | | 0.072 | 0.059 | 0.472 |
| M-2-6- | 8.1  (4.2-14.4) | | 5.8  (2.9-9.7) | | 0.050 | - | | - | | - | 18.9  (7.2-36.1) | | 17.0  (5.6-29.5) | | 0.728 | 8.0  (4.6-27.0) | | 14.9  (11.7-26.8) | | 0.149 | 0.107 | 0.856 |
|  |  | |  | |  |  | |  | |  |  | |  | |  |  | |  | |  |  |  |
|  | Gender | | | | | | | | | | | | | | | | | | | | | |
|  | female  (*n*=37) | | male  (*n*=64) | |  | female | | male | |  | female  (*n*=19) | | male  (*n*=22) | |  | female  (*n*=17) | | male  (*n*=19) | |  |  |  |
| Sub-population | median  (25^th^-75^th^ perc.) | | | | *p*-value* |  | |  | | *p*-value | median  (25^th^-75^th^ perc.) | | | | *p*-value* | median  (25^th^-75^th^ perc.) | | | | *p*-value* | pooled  *p*-value^#^ | adjusted  pooled  *p*-value^ |
| M+2+6+ | 16.1  (1.9-25.8) | | 12.1  (3.2-22.2) | | 0.434 | - | | - | | - | 10.7  (3.2-22.7) | | 16.0  (10.4-24.4) | | 0.614 | 5.6 (3.3-11.6) | | 7.1 (3.7-22.1) | | 0.397 | 0.610 | 1.000 |
| M+2+6- | 9.1  (2.8-15.1) | | 9.1  (2.9-12.8) | | 0.600 | - | | - | | - | 2.5  (1.6-5.1) | | 2.5  (1.4-6.6) | | 0.886 | 5.3 (2.1-11.0) | | 7.7 (2.8-10.4) | | 0.684 | 0.917 | 1.000 |
| M+2-6+ | 6.3  (4.3-10.5) | | 4.5  (2.2-11.9) | | 0.329 | - | | - | | - | 7.8  (3.0-17.6) | | 4.2  (1.3-10.5) | | 0.180 | 2.1 (1.5-12.8) | | 2.6 (1.6-4.8) | | 0.684 | 0.379 | 1.000 |
| M+2-6- | 5.4  (3.4-11.8) | | 4.3  (2.7-8.4) | | 0.173 | - | | - | | - | 3.8  (1.2-13.4) | | 1.6  (0.7-7.4) | | 0.344 | 3.5 (1.6-9.7) | | 3.4 (1.7-5.1) | | 0.616 | 0.358 | 1.000 |
| M-2+6+ | 19.9  (5.7-27.5) | | 21.5  (11.2-31.4) | | 0.350 | - | | - | | - | 17.0  (1.0-27.8) | | 15.4  (7.8-36.3) | | 0.509 | 17.2 (1.7-24.8) | | 17.3 (8.1-33.0) | | 0.531 | 0.581 | 1.000 |
| M-2+6- | 13.9  (7.2-21.7) | | 19.3  (10.5-26.4) | | 0.094 | - | | - | | - | 6.8  (1.1-12.9) | | 9.3  (5.1-18.4) | | 0.227 | 16.7 (3.9-31.9) | | 15.0 (10.6-23.8) | | 0.950 | 0.253 | 1.000 |
| M-2-6+ | 6.8  (3.7-17.7) | | 5.0  (3.0-14.3) | | 0.396 | - | | - | | - | 11.3  (6.8-20.3) | | 8.7  (4.0-17.7) | | 0.429 | 8.8 (5.8-16.8) | | 8.6 (5.7-16.8) | | 0.707 | 0.644 | 1.000 |
| M-2-6- | 6.3  (2.9-18.2) | | 6.4  (4.0-11.1) | | 0.992 | - | | - | | - | 22.7  (7.0-32.1) | | 11.7  (6.2-36.0) | | 0.786 | 12.2 (6.1-22.3) | | 14.2 (5.6-27.7) | | 1.000 | 0.998 | 1.000 |
|  |  | |  | |  |  | |  | |  |  | |  | |  |  | |  | |  |  |  |
|  | c-MYC translocation (FISH) | | | | | | | | | | | | | | | | | | | | | |
|  | negative  (*n*=87) | | positive  (*n*=13) | |  | negative | | positive | |  | negative | | positive | |  | negative  (*n*=26) | | positive  (*n*=8) | |  |  |  |
| Sub-population | median  (25^th^-75^th^ perc.) | | | | *p*-value* |  | |  | | *p*-value | median  (25^th^-75^th^ perc.) | | | | *p*-value | median  (25^th^-75^th^ perc.) | | | | *p*-value* | pooled  *p*-value^#^ | adjusted  pooled  *p*-value^ |
| M+2+6+ | 14.0  (3.1-22.9) | | 12.3  (0.4-35.1) | | 0.566 | - | | - | | - | - | | - | | - | 5.9  (2.9-11.0) | | 28.0  (13.2-32.5) | | 0.010 | 0.036 | 0.288 |
| M+2+6- | 9.1  (3.2-13.6) | | 5.3  (0.4-11.5) | | 0.616 | - | | - | | - | - | | - | | - | 5.4  (2.2-9.9) | | 9.6  (7.6-12.2) | | 0.130 | 0.282 | 1.000 |
| M+2-6+ | 4.8  (2.6-11.5) | | 6.7  (4.9-12.9) | | 0.247 | - | | - | | - | - | | - | | - | 2.1  (1.5-4.8) | | 6.4  (1.6-12.5) | | 0.220 | 0.213 | 1.000 |
| M+2-6- | 4.3  (2.9-9.0) | | 8.9  (3.9-11.5) | | 0.200 | - | | - | | - | - | | - | | - | 3.7  (1.3-8.0) | | 3.1  (1.9-8.0) | | 0.827 | 0.463 | 1.000 |
| M-2+6+ | 22.0  (11.1-31.5) | | 11.1  (0.3-19.9) | | 0.008 | - | | - | | - | - | | - | | - | 17.1  (6.5-28.6) | | 15.9  (7.7-26.4) | | 0.952 | 0.043 | 0.344 |
| M-2+6- | 16.9  (8.7-24.4) | | 10.8  (0.8-23.4) | | 0.260 | - | | - | | - | - | | - | | - | 18.1  (7.0-31.3) | | 12.4  (7.4-16.3) | | 0.327 | 0.294 | 1.000 |
| M-2-6+ | 6.5  (3.3-15.1) | | 4.0  (3.4-7.2) | | 0.330 | - | | - | | - | - | | - | | - | 9.3  (5.5-19.3) | | 7.5  (5.8-9.6) | | 0.347 | 0.363 | 1.000 |
| M-2-6- | 6.0  (3.5-12.8) | | 8.9  (5.2-11.8) | | 0.389 | - | | - | | - | - | | - | | - | 17.4  (10.2-34.0) | | 5.4  (4.4-10.7) | | 0.015 | 0.036 | 0.288 |
|  |  | |  | |  |  | |  | |  |  | |  | |  |  | |  | |  |  |  |
|  | IPI Risk Score | | | | | | | | | | | | | | | | | | | | | |
|  | Low  (*n*=32) | Med.  (*n*=40) | | High  (*n*=20) |  | Low  (*n*=31) | Med.  (*n*=31) | | High  (*n*=5) |  | Low  (*n*=17) | Med.  (*n*=16) | | High  (*n*=5) |  | Low  (*n*=13) | Med.  (*n*=13) | | High  (*n*=10) |  |  |  |
| Sub-population | median  (25^th^-75^th^ perc.) | | | | *p*-value^$^ | median  (25^th^-75^th^ perc.) | | | | *p*-value^$^ | median  (25^th^-75^th^ perc.) | | | |  | median  (25^th^-75^th^ perc.) | | | | *p*-value^$^ | pooled  *p*-value^#^ | adjusted  pooled  *p*-value^ |
| M+2+6+ | 9.7  (2.6-24.0) | 12.3  (2.6-18.7) | | 17.7  (9.7-27.7) | 0.192 | 5.4  (1.6-10.9) | 8.0  (2.5-19.0) | | 3.6  2.9-8.6) | 0.499 | 13.7  (5.4-20.3) | 13.2  (8.6-29.6) | | 18.3  (3.3-40.7) | 0.772 | 6.2  (4.5-22.2) | 6.2  (2.7-16.4) | | 6.4  (2.7-23.1) | 0.67 | 0.646 | 1.000 |
| M+2+6- | 6.2  (1.5-11.8) | 9.3  (3.2-14.4) | | 11.3  (4.6-15.2) | 0.34 | 6.9  (2.6-10.6) | 5.5  (1.4-9.5) | | 8.3  (7.9-10.0) | 0.565 | 2.6  (2.0-5.1) | 2.2  (1.1-14.1) | | 3.4  (1.4-4.3) | 0.997 | 7.4  (3.1-10.0) | 8.5  (2.4-9.9) | | 3.8  (1.5-12.9) | 0.946 | 0.906 | 1.000 |
| M+2-6+ | 4.8  (2.1-11.5) | 6.2  (2.6-12.9) | | 5.5  (3.7-10.8) | 0.902 | 7.4  (4.3-15.7) | 12.0  (6.3-20.8) | | 3.5  (2.8-5.4) | 0.214 | 5.5  (2.2-10.9) | 4.8  (1.3-10.2) | | 3.9  (2.1-22.3) | 0.774 | 2.6  (1.5-4.9) | 2.1  (1.5-2.7) | | 6.4  (2.5-17.2) | 0.139 | 0.458 | 1.000 |
| M+2-6- | 3.9  (2.4-7.6) | 6.1  (3.6-10.6) | | 4.1  (3.4-6.5) | 0.159 | 10.4  (6.5-14.9) | 9.1  (3.9-12.2) | | 13.3  (6.3-16.0) | 0.407 | 2.5  (1.2-8.6) | 2.3  (0.7-8.6) | | 0.7  (0.5-7.0) | 0.721 | 3.8  (2.8-5.6) | 2.4  (1.2-4.6) | | 4.9  (2.5-8.3) | 0.492 | 0.479 | 1.000 |
| M-2+6+ | 22.1  (7.6-29.1) | 17.6  (9.9-30.2) | | 24.2  (16.8-29.4) | 0.693 | 13.2  (4.6-31.9) | 13.9  (9.0-29.1) | | 19.0  (12.8-25.1) | 0.989 | 17.0 (1.7-36.4) | 15.4  (6.2-27.7) | | 18.8  (1.9-23.3) | 0.999 | 17.3  (8.1-22.6) | 23.1  (6.8-34.2) | | 9.1  (2.9-19.6) | 0.412 | 0.960 | 1.000 |
| M-2+6- | 19.0  (7.5-23.6) | 16.0  (7.6-23.0) | | 16.4  (9.8-22.4) | 0.924 | 14.1  (8.2-20.6) | 11.3  (5.9-15.9) | | 25.5  (16.2-28.5) | 0.057 | 11.0  (1.9-18.5) | 7.1  (5.3-15.8) | | 7.0  (1.0-13.6) | 0.71 | 15.0  (8.6-23.0) | 21.2  14.5-32.9) | | 10.3  (2.0-22.0) | 0.363 | 0.377 | 1.000 |
| M-2-6+ | 6.9  (2.9-13.9) | 6.4  (3.5-18.7) | | 4.2  (2.6-7.3) | 0.337 | 9.0  (4.6-20.1) | 11.3  (6.0-20.9) | | 5.8  (5.8-15.0) | 0.415 | 11.3  (6.1-18.9) | 8.0  (2.8-17.5) | | 8.6  (6.6-19.0) | 0.893 | 9.5  (4.4-17.5) | 8.2  (6.0-12.4) | | 11.0  (7.0-16.4) | 0.939 | 0.830 | 1.000 |
| M-2-6- | 5.8  (3.9-11.4) | 9.0  (3.9-17.7) | | 5.6  (2.7-8.1) | 0.152 | 6.8  (3.9-13.5) | 9.3  (2.8-12.8) | | 3.9  (3.8-15.2) | 0.993 | 17.0  (5.6-36.0) | 14.2  (6.6-31.3) | | 10.2  (2.6-29.5) | 0.642 | 12.6  (9.6-25.9) | 14.1  (6.4-24.1) | | 12.6  (4.9-27.6) | 0.982 | 0.789 | 1.000 |
|  | | | | | | | | | | | | | | | | | | | | | | |
| *Mann-Whitney test  ^#^Fisher's method  ^Bonferroni correction  ^$^Kruskal–Wallis test  perc.- percentile, FISH - fluorescence *in situ* hybridization, med. – medium, IPI Risk Group - International Prognostic Index Risk Group, NUH – National University Hospital, CMMC - Chi-Mei Medical Center, SGH – Singapore General Hospital, MDA – MD Anderson Cancer Center. | | | | | | | | | | | | | | | | | | | | | | |

| Supplementary table 4. Pooled univariate analysis for MYC, BCL2 and BCL6 single oncogene and sub-populations percentage extents as a continuous variable at 5% increments as predictors for overall survival (OS) in mfIHC cohorts of DLBCL (Cox proportional hazards model). | | | | | | | | | | | | |
| --- | --- | --- | --- | --- | --- | --- | --- | --- | --- | --- | --- | --- |
|  | | | | | | | | | | | | |
|  | NUH  (*n*=98) | | SGH  (*n*=41) | | MDA  (*n*=36) | | pooled | | | | | |
|  |  |  |  |  |  |  |  | random-effects weighting | | |  |  |
|  | HR  (95% CI) | *p-value* | HR  (95% CI) | *p-*value | HR  (95% CI) | *p-*value | HR  (95% CI) | NUH | SGH | MDA | *p-*value | adjusted *p*-value^#^ |
| Single oncogene | | | | | | | | | | | | |
| MYC | 0.97  (0.88-1.1) | 0.586 | 1.1  (0.96-1.4) | 0.130 | 1.2  (1.0-1.4) | 0.217 | 1.1  (0.95-1.2) | 0.41 | 0.29 | 0.30 | 0.217 | 0.650 |
| BCL2 | 1.1  (1.0-1.2) | 0.044 | 1.1  (1.0-1.2) | 0.059 | 1.0  (0.91-1.1) | 0.974 | 1.1  (1.0-1.1) | 0.39 | 0.38 | 0.23 | 0.017 | 0.051 |
| BCL6 | 0.92  (0.83-1.0) | 0.133 | 1.1  (0.95-1.3) | 0.185 | 0.99 (0.89-1.1) | 0.890 | 0.99  (0.89-1.1) | 0.42 | 0.28 | 0.30 | 0.890 | 1.000 |
|  |  |  |  |  |  |  |  |  |  |  |  |  |
| Sub-populations | | | | | | | | | | | | |
| M+2+6+ | 1.0  (0.90-1.2) | 0.728 | 1.3  (1.1-1.5) | 0.009 | 1.1  (0.89-1.4) | 0.319 | 1.1  (0.98-1.3) | 0.46 | 0.33 | 0.21 | 0.085 | 0.677 |
| M+2+6- | 1.3  (1.1-1.5) | 0.007 | 1.6  (1.1-2.5) | 0.019 | 1.5  (1.1-2.0) | 0.007 | 1.4  (1.2-1.6) | 0.65 | 0.12 | 0.23 | 1.7x10^-5^ | 0.0001 |
| M+2-6+ | 0.79  (0.61-1.0) | 0.083 | 0.88  (0.65-1.2) | 0.373 | 1.1  (0.88-1.5) | 0.311 | 0.93  (0.74-1.2) | 0.35 | 0.31 | 0.34 | 0.503 | 1.000 |
| M+2-6- | 0.90  (0.63-1.3) | 0.543 | 0.90  (0.68-1.2) | 0.527 | 1.4  (0.97-2.0) | 0.074 | 1.0  (0.78-1.4) | 0.32 | 0.37 | 0.31 | 0.824 | 1.000 |
| M-2+6+ | 1.0  (0.96-1.2) | 0.192 | 1.0  (0.91-1.2) | 0.554 | 0.89  (0.73-1.1) | 0.267 | 1.0  (0.92-1.1) | 0.41 | 0.37 | 0.22 | 0.641 | 1.000 |
| M-2+6- | 1.2  (1.0-1.4) | 0.010 | 1.2  (0.84-1.6) | 0.367 | 0.93  (0.74-1.2) | 0.529 | 1.1  (0.94-1.3) | 0.49 | 0.20 | 0.31 | 0.234 | 1.000 |
| M-2-6+ | 0.90  (0.75-1.1) | 0.265 | 0.89  (0.68-1.2) | 0.369 | 0.94  (0.68-1.3) | 0.686 | 0.90 (0.79-1.0) | 0.55 | 0.27 | 0.18 | 0.144 | 1.000 |
| M-2-6- | 0.86  (0.70-1.1) | 0.171 | 0.85  (0.72-1.0) | 0.061 | 0.92  (0.74-1.1) | 0.420 | 0.87  (0.78-0.98) | 0.29 | 0.44 | 0.27 | 0.016 | 0.131 |
|  | | | | | | | | | | | | |
| ^#^Bonferroni correction  HR – hazard ratio for death, 95% CI – 95% confidence interval. | | | | | | | | | | | | |

| Supplementary table 5. Univariate analysis of clinicopathological features as a predictor of overall survival (OS) after first-line R-CHOP treatment in the NUH, SGH and MDA cohorts of DLBCL (Cox proportional hazards model). | | | | | | |
| --- | --- | --- | --- | --- | --- | --- |
|  | NUH | | SGH | | MDA | |
|  | total cases *n*=90  missing values *n*=8 | | total cases *n*=37  missing values *n*=3 | | total cases *n*=36 | |
|  | HR (95% CI) | *p-*value | HR (95% CI) | *p-*value | HR (95% CI) | *p-*value |
|  |  |  |  |  |  |  |
| IPI Risk Group |  | 0.410 |  | 0.019 |  | 0.294 |
| Low | Ref. |  | Ref. |  | Ref. |  |
| Intermediate | 1.5 (0.60 to 3.7) | 0.401 | 6.1 (1.3 to 28.4) | 0.021 | 1.1 (0.22 to 5.5) | 0.914 |
| High | 1.9 (0.73 to 5.2) | 0.184 | 11.3 (2.1 to 61.8) | 0.005 | 2.7 (0.64 to 11.4) | 0.176 |
|  |  |  |  |  |  |  |
|  |  |  |  |  |  |  |
|  | total cases *n*=94  missing values *n*=4 | | - | | total cases *n*=34  missing values *n*=2 | |
|  | HR (95% CI) | *p-*value | - | *-* | HR (95% CI) | *p-*value |
|  |  |  |  |  |  |  |
| c-MYC translocation status |  | 0.309 |  | - |  | 0.098 |
| negative | Ref. |  | - |  | Ref. |  |
| positive | 0.47 (0.11 to 2.0) |  | - | - | 3.1 (0.81 to 11.5) |  |
|  |  |  |  |  |  |  |
| IPI Risk Group - International Prognostic Index Risk Group, NUH – National University Hospital, SGH – Singapore General Hospital, MDA – MD Anderson Cancer Center, 95% CI – 95% confidence interval, Ref. – reference group. | | | | | | |

Supplementary table 6 – in a separate Excel file

**Supplementary table 6.** Inferred percentage extents of MYC, BCL2, BCL6 and sub-population metrics in GEP cohorts.

| Supplementary table 7. Pooled univariate analysis for sub-population metrics as a continuous variable at 5% increments as predictors for overall survival (OS) in GEP DLBCL cohorts (Cox proportional hazards model). | | | | | | | | | | | | | | | | | | | | | | | | | | | |
| --- | --- | --- | --- | --- | --- | --- | --- | --- | --- | --- | --- | --- | --- | --- | --- | --- | --- | --- | --- | --- | --- | --- | --- | --- | --- | --- | --- |
|  | | | | | | | | | | | | | | | | | | | | | | | | | | | |
|  | Reddy *et al.*  *n*=753 | | Visco *et al.*  *n*=470 | | Sha *et al.*  *n*=469 | | Schmitz *et al.*  *n*=234 | | Lenz *et al.*  *n*=232 | | Barrans *et al.*  *n*=140 | | Dubois *et al.*  *n*=122 | | Chapuy *et al.*  *n*=101 | | pooled | | | | | | | | | | |
|  |  |  |  |  |  |  |  |  |  |  |  |  |  |  |  |  |  | random-effects weighting | | | | | | | |  |  |
|  | HR  (95% CI) | *p-value* | HR  (95% CI) | *p-value* | HR  (95% CI) | *p-value* | HR  (95% CI) | *p-value* | HR  (95% CI) | *p-value* | HR  (95% CI) | *p-value* | HR  (95% CI) | *p-value* | HR  (95% CI) | *p-value* | HR  (95% CI) | *Reddy et al.* | *Visco et al.* | *Sha et al.* | Schmitz *et al.* | *Lenz et al.* | *Barrans et al.* | *Dubois et al.* | *Chapuy et al.* | *p-*value | adjusted *p*-value^#^ |
| Sub-populations | | | | | | | | | | | | | | | | | | | | | | | | | | | |
| M+2+6+ | 1.1 (1.0-1.1) | 0.045 | 1.1 (0.99-1.1) | 0.089 | 1.1 (1.0-1.1) | 0.007 | 1.1 (1.0-1.2) | 0.055 | 1.00 (0.93-1.1) | 0.952 | 1.1 (1.0-1.3) | 0.039 | 1.1 (0.94-1.2) | 0.345 | 1.1 (0.96-1.3) | 0.125 | 1.1 (1.03-1.1) | 0.22 | 0.16 | 0.26 | 0.1 | 0.14 | 0.05 | 0.04 | 0.03 | 7.3 x 10^-6^ | 5.8 x 10^-5^ |
| M+2+6- | 1.1 (1.1-1.2) | 5.5 x 10^-6^ | 1.1 (1.1-1.2) | 3.5 x 10^-6^ | 1.2 (1.1-1.3) | 2.0 x 10^-8^ | 1.2 (1.1-1.3) | 6.4 x 10^-5^ | 1.2 (1.1-1.2) | 1.4 x 10^-8^ | 1.1 (1.0-1.2) | 0.009 | 1.2 (1.1-1.4) | 2.6 x 10^-4^ | 1.2 (1.1-1.3) | 0.003 | 1.2 (1.1-1.2) | 0.27 | 0.17 | 0.15 | 0.08 | 0.17 | 0.07 | 0.04 | 0.04 | 3.1 x 10^-33^ | 2.4 x 10^-32^ |
| M+2-6+ | 0.95 (0.88-1.0) | 0.176 | 0.93 (0.85-1.0) | 0.118 | 0.97 (0.86-1.1) | 0.553 | 0.83 (0.72-0.96) | 0.011 | 0.96 (0.87-1.1) | 0.467 | 1.0 (0.88-1.2) | 0.823 | 0.88 (0.71-1.1) | 0.245 | 1.1 (0.92-1.2) | 0.375 | 0.95 (0.91-1.0) | 0.24 | 0.18 | 0.13 | 0.09 | 0.16 | 0.09 | 0.04 | 0.07 | 0.020 | 0.162 |
| M+2-6- | 1.1 (0.97-1.1) | 0.197 | 1.0 (0.92-1.1) | 0.719 | 1.0 (0.89-1.1) | 0.923 | 1.1 (0.96-1.2) | 0.185 | 1.2 (1.1-1.3) | 2.7 x 10^-5^ | 1.1 (0.96-1.3) | 0.153 | 0.99 (0.82-1.2) | 0.886 | 1.2 (0.90-1.5) | 0.258 | 1.1 (1.03-1.1) | 0.21 | 0.16 | 0.11 | 0.12 | 0.24 | 0.07 | 0.06 | 0.03 | 0.001 | 0.009 |
| M-2+6+ | 0.98 (0.93-1.0) | 0.391 | 0.98 (0.93-1.0) | 0.539 | 0.98 (0.93-1.0) | 0.579 | 0.94 (0.88-1.0) | 0.091 | 0.86 (0.81-0.91) | 6.6 x 10^-7^ | 0.93 (0.84-1.0) | 0.134 | 0.95 (0.85-1.1) | 0.318 | 0.94 (0.82-1.1) | 0.333 | 0.95 (0.92-1.0) | 0.18 | 0.16 | 0.15 | 0.13 | 0.15 | 0.09 | 0.08 | 0.05 | 0.002 | 0.016 |
| M-2+6- | 1.0 (0.97-1.1) | 0.405 | 1.1 (1.0-1.1) | 5.9 x 10^-4^ | 0.98 (0.92-1.0) | 0.553 | 1.1 (1.00-1.2) | 0.054 | 1.0 (0.98-1.1) | 0.169 | 0.93 (0.84-1.0) | 0.15 | 1.1 (0.97-1.2) | 0.136 | 0.97 (0.85-1.1) | 0.643 | 1.0 (0.99-1.1) | 0.18 | 0.18 | 0.14 | 0.12 | 0.15 | 0.09 | 0.07 | 0.06 | 0.134 | 1.000 |
| M-2-6+ | 0.90 (0.85-0.96) | 4.5 x 10^-4^ | 0.85 (0.79-0.91) | 7.8 x 10^-6^ | 0.88 (0.82-0.95) | 0.002 | 0.89 (0.82-0.96) | 0.003 | 0.90 (0.84-0.97) | 0.003 | 1.00 (0.90-1.1) | 0.937 | 0.81 (0.67-0.98) | 0.028 | 0.84 (0.70-1.00) | 0.048 | 0.89 (0.86-0.92) | 0.22 | 0.16 | 0.15 | 0.14 | 0.17 | 0.11 | 0.03 | 0.04 | 1.6 x 10^-10^ | 1.3 x 10^-9^ |
| M-2-6- | 0.95 (0.89-1.0) | 0.116 | 0.91 (0.84-0.99) | 0.024 | 0.90 (0.83-0.97) | 0.006 | 1.0 (0.97-1.1) | 0.269 | 1.0 (0.95-1.1) | 0.739 | 0.91 (0.78-1.1) | 0.201 | 0.93 (0.79-1.1) | 0.325 | 0.74 (0.56-0.96) | 0.026 | 0.95 (0.90-1.00) | 0.17 | 0.15 | 0.15 | 0.17 | 0.17 | 0.08 | 0.08 | 0.03 | 0.041 | 0.324 |
|  |  |  |  |  |  |  |  |  |  |  |  |  |  |  |  |  |  |  |  |  |  |  |  |  |  |  |  |
| ^#^Bonferroni correction  HR – hazard ratio for death, 95% CI – 95% confidence interval. | | | | | | | | | | | | | | | | | | | | | | | | | | | |

| Supplementary table 8. Multivariate analysis of continuous M+2+6- metric at 5% increments as a predictor of overall survival (OS) in cohorts with gene-expression data (Cox proportional hazards model). | | | | | | | | | | | | | | | | |
| --- | --- | --- | --- | --- | --- | --- | --- | --- | --- | --- | --- | --- | --- | --- | --- | --- |
|  | Reddy *et al.* | | Visco *et al.* | | Sha *et al.* | | Schmitz *et al.* | | Lenz *et al.* | | Barrans *et al.* | | Dubois *et al.* | | Chapuy *et al.* | |
|  | total cases *n*=616  missing values *n*=137 | | total cases *n*=424  missing values *n*=46 | | total cases *n*=459  missing values *n*=10 | | total cases *n*=200  missing values *n*=34 | | total cases *n*=163  missing values *n*=69 | | total cases *n*=140 | | total cases *n*=122 | | total cases *n*=97  missing values *n*=4 | |
|  | HR (95% CI) | *p-*value | HR (95% CI) | *p-*value | HR (95% CI) | *p-*value | HR (95% CI) | *p-*value | HR (95% CI) | *p-*value | HR (95% CI) | *p-*value | HR (95% CI) | *p-*value | HR (95% CI) | *p-*value |
| Sub-population |  |  |  |  |  |  |  |  |  |  |  |  |  |  |  |  |
| M+2+6- (continuous, per 5% of metric) | 1.12 (1.05 to 1.19) | <0.001 | 1.10 (1.03 to 1.17) | 0.005 | 1.18 (1.07 to 1.30) | 0.001 | 1.08 (0.96 to 1.21) | 0.198 | 1.11 (1.0 to 1.3) | 0.068 | 1.15 (1.04 to 1.28) | 0.008 | 1.18 (1.05 to 1.33) | 0.007 | 1.22 (1.06 to 1.4) | 0.007 |
|  |  |  |  |  |  |  |  |  |  |  |  |  |  |  |  |  |
| IPI Risk Group |  | <0.001 |  | <0.001 |  | <0.001 |  | <0.001 |  | <0.001 |  | - |  | 0.011 |  | 0.001 |
| Low | Ref. |  | Ref. |  | Ref. |  | Ref. |  | Ref. |  | - | - | Ref. |  | Ref. |  |
| Intermediate | 2.8 (1.8 to 4.2) | <0.001 | 2.8 (1.9 to 4.3) | <0.001 | 1.7 (0.90 to 3.3) | 0.103 | 1.9 (1.1 to 3.2) | 0.023 | 1.4 (0.61 to 3.2) | 0.429 | - | - | 2.7 (0.62 to 11.7) | 0.189 | 4.6 (1.3 to 15.8) | 0.016 |
| High | 5.7 (3.6 to 9.1) | <0.001 | 5.7 (3.5 to 9.2) | <0.001 | 4.0 (2.0 to 8.0) | <0.001 | 5.9 (3.1 to 11.4) | <0.001 | 5.2 (2.1 to 12.7) | <0.001 | - | - | 5.5 (1.3 to 24.7) | 0.019 | 11.2 (3.1 to 40.3) | <0.001 |
|  |  |  |  |  |  |  |  |  |  |  |  |  |  |  |  |  |
| Cell-of-origin (GEP) |  | 0.477 |  | 0.113 |  | 0.957 |  | 0.022 |  | 0.011 |  | 0.016 |  | 0.667 |  | 0.441 |
| GCB | Ref. |  | Ref. |  | Ref. |  | Ref. |  | Ref. |  | Ref. |  | Ref. |  | Ref. |  |
| ABC | 1.1 (0.77 to 1.6) | 0.575 | 1.3 (0.91 to 1.9) | 0.147 | 1.1 (0.62 to 1.9) | 0.798 | 2.2 (1.2 to 4.0) | 0.007 | 3.6 (1.5 to 8.8) | 0.005 | 1.4 (0.73 to 2.4) | 0.365 | 1.4 (0.64 to 3.2) | 0.377 | 0.57 (0.24 to 1.3) | 0.201 |
| UNC | 1.3 (0.85 to 2.0) | 0.224 | 1.7 (0.97 to 3.0) | 0.063 | 1.0 (0.56 to 1.8) | 0.973 | 1.8 (0.98 to 3.3) | 0.056 | 1.2 (0.42 to 4.8) | 0.571 | 0.40 (0.18 to 0.92) | 0.031 | 1.2 (0.42 to 3.2) | 0.785 | undefined | 0.968 |
|  | | | | | | | | | | | | | | | | |
| IPI Risk Group - International Prognostic Index Risk Group, GEP – gene-expression profiling, GCB - Germinal center B-cell-like diffuse large B-cell lymphoma, ABC - Activated B-cell lymphoma, UNC – unclassified, 95% CI – 95% confidence interval, Ref. – reference group. | | | | | | | | | | | | | | | | |

| Supplementary table 9. Univariate and multivariate analysis of continuous M+2+6- metric as a continuous variable at 5% increments as predictor of progression-free survival (PFS) and overall survival (OS) in the GOYA trial cohort (Cox proportional hazards model). | | | | | |
| --- | --- | --- | --- | --- | --- |
|  | | | | | |
| *univariate* | | | | | |
|  |  | |  | |  |
|  | PFS | | OS | |  |
|  | Total cases *n*=549 | | Total cases *n*=549 | |  |
|  | HR (95% CI) | *p-*value | HR (95% CI) | *p-*value |  |
| Sub-population |  |  |  |  |  |
| M+2+6- (continuous, per 5% of metric) | 1.03 (1.01 to 1.04) | 0.0001 | 1.03 (1.01 to 1.04) | 0.0004 |  |
|  |  |  |  |  |  |
|  |  |  |  |  |  |
| *multivariate* | | | | |  |
|  | | | | |  |
|  | PFS | | OS | |  |
|  | Total cases *n*=549 | | Total cases *n*=549 | |  |
|  | HR (95% CI) | *p-*value | HR (95% CI) | *p-*value |  |
| Sub-population |  |  |  |  |  |
| M+2+6- (continuous, per 5% of metric) | 1.02 (1.00 to 1.03) | 0.021 | 1.01 (0.998 to 1.03) | 0.089 |  |
|  |  |  |  |  |  |
| IPI Risk Group |  |  |  |  |  |
| Low | Ref. |  | Ref. |  |  |
| Intermediate | 1.4 (0.90 to 2.3) | 0.133 | 1.4 (0.76 to 2.6) | 0.277 |  |
| High | 3.1 (1.8 to 5.3) | < 0.0001 | 3.9 (2.0 to 7.4) | < 0.0001 |  |
|  |  |  |  |  |  |
| Cell-of-origin (GEP) |  |  |  |  |  |
| GCB | Ref. |  | Ref. |  |  |
| ABC | 1.2 (0.70 to 2.0) | 0.522 | 1.4 (0.73 to 2.5) | 0.336 |  |
| UNC | 1.2 (0.53 to 2.6) | 0.695 | 1.0 (0.39 to 2.8) | 0.929 |  |
| unknown | 1.2 (0.79 to 1.9) | 0.370 | 1.1 (0.70 to 2.0) | 0.621 |  |
|  |  |  |  |  |  |
| IPI Risk Group - International Prognostic Index Risk Group, GEP – gene expression profiling, GCB - germinal center B-cell-like diffuse large B-cell lymphoma, ABC - activated B-cell diffuse large B-cell Lymphoma, UNC – unclassified, 95% CI – 95% confidence interval, Ref. – reference group. | | | | | |

| Supplementary table 10. Multivariate analysis of M+2+6- metric dichotomized at 15% as a predictor of overall survival (OS) in cohorts with gene-expression data (Cox proportional hazards model). | | | | | | | | | | | | | | | | |
| --- | --- | --- | --- | --- | --- | --- | --- | --- | --- | --- | --- | --- | --- | --- | --- | --- |
|  | Reddy *et al.* | | Visco *et al.* | | Sha *et al.* | | Schmitz *et al.* | | Lenz *et al.* | | Barrans *et al.* | | Dubois *et al.* | | Chapuy *et al.* | |
|  | total cases *n*=616  missing values *n*=137 | | total cases *n*=424  missing values *n*=46 | | total cases *n*=459  missing values *n*=10 | | total cases *n*=200  missing values *n*=34 | | total cases *n*=164  missing values *n*=69 | | total cases *n*=140 | | total cases *n*=122 | | total cases *n*=97  missing values *n*=4 | |
|  | HR (95% CI) | *p-*value | HR (95% CI) | *p-*value | HR (95% CI) | *p-*value | HR (95% CI) | *p-*value | HR (95% CI) | *p-*value | HR (95% CI) | *p-*value | HR (95% CI) | *p-*value | HR (95% CI) | *p-*value |
| Sub-population |  | 0.035 |  | 0.052 |  | 0.060 |  | 0.632 |  | 0.068 |  | 0.024 |  | 0.006 |  | 0.007 |
| M+2+6- <15% | Ref. |  | Ref. |  | Ref. |  | Ref. |  | Ref. |  | Ref. |  | Ref. |  | Ref. |  |
| M+2+6- ≥15% | 1.4 (1.0 to 2.0) |  | 1.4 (1.0 to 2.1) |  | 1.6 (0.99 to 2.6) |  | 1.1 (0.67 to 2.0) |  | 1.9 (0.95 to 3.9) |  | 1.9 (1.1 to 3.3) |  | 2.6 (1.3 to 5.2) |  | 3.3 (1.4 to 7.7) |  |
|  |  |  |  |  |  |  |  |  |  |  |  |  |  |  |  |  |
| IPI Risk Group |  | <0.001 |  | <0.001 |  | <0.001 |  | <0.001 |  | <0.001 |  | - |  | 0.007 |  | 0.001 |
| Low | Ref. |  | Ref. |  | Ref. |  | Ref. |  | Ref. |  | - | - | Ref. |  | Ref. |  |
| Intermediate | 2.6 (1.7 to 4.0) | <0.001 | 2.8 (1.9 to 4.4) | <0.001 | 1.7 (0.91 to 3.3) | 0.098 | 1.8 (1.1 to 3.2) | 0.029 | 1.2 (0.56 to 3.0) | 0.684 | - | - | 2.5 (0.57 to 10.8) | 0.224 | 4.7 (1.4 to 16.4) | 0.014 |
| High | 5.4 (3.4 to 8.6) | <0.001 | 5.7 (3.5 to 9.3) | <0.001 | 4.1 (2.1 to 8.1) | <0.001 | 5.8 (3.0 to 11.1) | <0.001 | 4.7 (1.9 to 11.6) | <0.001 | - | - | 5.8 (1.3 to 24.8) | 0.019 | 11.0 (3.1 to 39.8) | <0.001 |
|  |  |  |  |  |  |  |  |  |  |  |  |  |  |  |  |  |
| Cell-of-origin (GEP) |  | 0.282 |  | 0.093 |  | 0.708 |  | 0.005 |  | 0.017 |  | 0.019 |  | 0.740 |  | 0.457 |
| GCB | Ref. |  | Ref. |  | Ref. |  | Ref. |  | Ref. |  | Ref. |  | Ref. |  | Ref. |  |
| ABC | 1.2 (0.85 to 1.8) | 0.268 | 1.4 (0.96 to 2.0) | 0.081 | 1.3 (0.73 to 2.2) | 0.408 | 2.5 (1.4 to 4.3) | 0.001 | 3.5 (1.4 to 8.7) | 0.006 | 1.4 (0.79 to 2.5) | 0.252 | 1.4 (0.61 to 3.1) | 0.439 | 0.57 (0.24 to 1.4) | 0.211 |
| UNC | 1.4 (0.91 to 2.1) | 0.129 | 1.7 (0.95 to 2.9) | 0.077 | 1.1 (0.60 to 1.9) | 0.818 | 1.9 (1.0 to 3.5) | 0.035 | 1.5 (0.44 to 4.9) | 0.541 | 0.44 (0.20 to 0.98) | 0.044 | 1.2 (0.44 to 3.4) | 0.689 | undefined | 0.968 |
|  | | | | | | | | | | | | | | | | |
| IPI Risk Group - International Prognostic Index Risk Group, GEP – gene-expression profiling, GCB - Germinal center B-cell-like diffuse large B-cell lymphoma, ABC - Activated B-cell lymphoma, UNC – unclassified, 95% CI – 95% confidence interval, Ref. – reference group. | | | | | | | | | | | | | | | | |

Supplementary table 11 – in a separate Excel file

**Supplementary table 11**. Correlation of M+2+6- metric with gene expression in GEP cohorts.

Supplementary table 12 – in a separate Excel file

**Supplementary table 12**. Differential gene expression analysis of primary germinal center (GC) B-cells with M+2+ and M+2+6+ overexpression.

Supplementary table 13 – in a separate Excel file

**Supplementary table 13**. Differentially expressed genes between M+2+6- and all other malignant cells in scRNA-seq samples of DLBCL. Dichotomized non-parametric comparison, Wilcoxon rank sum test.

Supplementary table 14 – in a separate Excel file

**Supplementary table 14**. Analysis of positive enrichment of Wikipathways terms between M+2+6- and all other malignant cells in scRNA-seq samples of DLBCL by gprofiler2.

| Supplementary table 15. Clinicopathologic characteristics of DLBCL patients evaluated by multiplexed fluorescent immunohistochemistry (mfIHC) in this study. | | | | | | | | | | |
| --- | --- | --- | --- | --- | --- | --- | --- | --- | --- | --- |
| Cohort | NUH | | CMMC | | SGH | | MDA | | BCA | |
|  | Total cases | Survival analysis | Total cases | Survival analysis | Total cases | Survival analysis | Total cases | Survival analysis | Total cases | Survival analysis |
|  | *n* (%) | *n* (%) | *n* (%) | *n* (%) | *n* (%) | *n* (%) | *n* (%) | *n* (%) | *n* (%) | *n* (%) |
| Number of patients | 152 (100%) | 98  (100%) | 150 (100%) | 0 | 67  (100%) | 41  (100%) | 40  (100%) | 36  (100%) | 303  (100%) | 274  (100%) |
| Age |  |  |  |  |  |  |  |  |  |  |
| ≤60 | 59 (38.8%) | 40 (40.8%) | 0 (0%) | - | 20 (29.9%) | 20 (48.8%) | 16 (40.0%) | 16 (44.4%) | 114 (37.6%) | 111 (40.5%) |
| >60 | 70 (46.1%) | 58 (59.2%) | 0 (0%) | - | 21 (31.3%) | 21 (51.2%) | 20 (50.0%) | 20 (55.6%) | 165 (54.5%) | 163 (59.5%) |
| no data | 23 (15.1%) | - | 150 (100%) | - | 26 (38.8%) | - | 4 (10.0%) | - | 24 (7.9%) | - |
|  |  |  |  |  |  |  |  |  |  |  |
| Gender |  |  |  |  |  |  |  |  |  |  |
| Female | 46 (30.3%) | 35 (35.7%) | 0 (0%) | - | 19 (28.4%) | 19 (46.3%) | 17 (42.5%) | 17 (47.2%) | 100 (33.0%) | 100 (36.5%) |
| Male | 83 (54.6%) | 63 (64.3%) | 0 (0%) | - | 22 (32.8%) | 22 (53.7%) | 19 (47.5%) | 19 (52.8%) | 174 (57.4%) | 174 (63.5%) |
| no data | 23 (15.1%) | - | 150 (100%) | - | 26 (38.8%) | - | 4 (10.0%) | - | 29 (9.6%) | - |
|  |  |  |  |  |  |  |  |  |  |  |
| IPI Risk Group |  |  |  |  |  |  |  |  |  |  |
| Low | 38 (25.0%) | 31 (31.6%) | 31 (20.7%) | - | 17 (25.4%) | 17 (41.5%) | 13 (32.5%) | 13 (36.1%) | 90 (29.7%) | 87 (31.8%) |
| Intermediate | 53 (34.9%) | 39 (39.8%) | 31 (20.7%) | - | 16 (23.9%) | 16 (39.0%) | 13 (32.5%) | 13 (36.1%) | 118 (38.9%) | 117 (42.7%) |
| High | 22 (14.5%) | 20 (20.4%) | 5 (3.3%) | - | 5 (7.5%) | 5 (12.2%) | 10 (25.0%) | 10 (27.8%) | 47 (15.4%) | 46 (16.8%) |
| no data | 39 (25.6%) | 8 (8.2%) | 83 (55.3%) | - | 29 (43.3%) | 3 (7.3%) | 4 (10.0%) | - | 48 (15.8%) | 24 (8.8%) |
|  |  |  |  |  |  |  |  |  |  |  |
| Cell-of-origin (Hans) |  |  |  |  |  |  |  |  |  |  |
| GC | 48 (31.6%) | 42 (42.9%) | 36 (24.0%) | - | 0 (0%) | 0 (0%) | 20 (50.0%) | 20 (55.5%) | 166 (54.8%) | 164 (59.9%) |
| non-GC | 48 (31.6%) | 43(43.9%) | 91 (60.7%) | - | 0 (0%) | 0 (0%) | 15 (37.5%) | 15 (41.7%) | 110 (36.3%) | 109 (39.8%) |
| no data | 56 (36.8%) | 13 (13.3%) | 23 (15.3%) | - | 67 (100%) | 41 (100%) | 5 (12.5%) | 1 (2.8%) | 27 (8.9%) | 1 (0.4%) |
|  |  |  |  |  |  |  |  |  |  |  |
| c-MYC translocation status |  |  |  |  |  |  |  |  |  |  |
| Negative | 124 (81.6%) | 81 (82.7%) | 0 (0%) | - | 31 (46.3%) | not included (little positive cases) | 26 (65.0%) | 26 (72.2%) | 228 (75.2%) | 228 (83.2%) |
| Positive | 19 (12.5%) | 13 (13.3%) | 0 (0%) | - | 2 (3.0%) |  | 8 (20.0%) | 8 (22.2%) | 41 (13.5%) | 41 (15.0%) |
| no data | 9 (5.9%) | 4 (4.1%) | 150 (100%) | - | 34 (50.7%) |  | 6 (15.0%) | 2 (5.6%) | 34 (11.2%) | 5 (1.8%) |
|  |  |  |  |  |  |  |  |  |  |  |
| BCL2 translocation status |  |  |  |  |  |  |  |  |  |  |
| Negative | 16 (10.5%) | 11 (11.2%) | 0 (0%) | - | - | - | - | - | 175 (57.8%) | 175 (63.9%) |
| Positive | 3 (2.0%) | 2 (2.1%) | 0 (0%) | - | - | - | - | - | 77 (25.4%) | 77 (28.1%) |
| no data | 133 (87.5%) | 85 (86.7%) | 150 (100%) | - | - | - | - | - | 51 (16.8%) | 22 (8.0%) |
|  |  |  |  |  |  |  |  |  |  |  |
| BCL6 translocation status |  |  |  |  |  |  |  |  |  |  |
| Negative | 13 (8.6%) | 8 (8.2%) | 0 (0%) | - | - | - | - | - | 203 (67.0%) | 203 (74.1%) |
| Positive | 6 (3.9%) | 5 (5.1%) | 0 (0%) | - | - | - | - | - | 55 (18.2%) | 55 (20.1%) |
| no data | 133 (87.5%) | 85 (86.7%) | 150 (100%) | - | - | - | - | - | 45 (14.9%) | 16 (5.8%) |
|  |  |  |  |  |  |  |  |  |  |  |
| Biopsy |  |  |  |  |  |  |  |  |  |  |
| Primary | 104 (68.4%) | 98 (100%) | - | - | 67 (100%) | 41 (100%) | 40 (100%) | 36 (100%) | 303 (100%) | 274 (100%) |
| Relapsed | 28 (18.4%) | - | - | - | - | - | - | - | - | - |
| no data | 20 (13.2%) | - | 150 (100%) | - | - | - | - | - | - | - |
|  |  |  |  |  |  |  |  |  |  |  |
| IPI Risk Group - International Prognostic Index Risk Group, GC - Germinal center B-cell-like diffuse large B-cell lymphoma, NUH – National University Hospital, CMMC - Chi-Mei Medical Center, SGH – Singapore General Hospital, MDA – MD Anderson Cancer Center, BCA - British Columbia Cancer Agency. | | | | | | | | | | |

| Supplementary table 16. Manual multiplexed fluorescent immunohistochemistry (mfIHC) staining protocol performed on the NUH and CMMC cohort TMA. | | |
| --- | --- | --- |
| Procedure | Reagent (product; dilution) | Duration (min.) |
| 1^st^ primary antibody incubation | anti-Bcl-6 (Leica, LN22, NCL-564; 1:30) | 60 |
| 1^st^ secondary antibody incubation | anti-Mouse HRP (Agilent Cat# K4001, RRID: AB_2827819) | 10 |
| 1^st^ signal amplification and fluorophore deposition | TSA Opal 670 (PerkinElmer FP1487001KT; 1:100) | 10 |
| 2^nd^ epitope retrieval (antibody stripping) | pH 9 HIER (low power) | 10 |
| 2^nd^ epitope blocking | BSA | 10 |
| 2^nd^ primary antibody incubation | anti-Bcl-2 (Agilent Cat# M0887, RRID: AB_2064429; 1:50) | 60 |
| 2^nd^ secondary antibody incubation | anti-Mouse HRP | 10 |
| 2^nd^ signal amplification and fluorophore deposition | TSA Opal 520 (PerkinElmer FP1487001KT; 1:100) | 10 |
| 3^rd^ epitope retrieval (antibody stripping) | pH 9 HIER (low power) | 10 |
| 3^rd^ epitope blocking | BSA | 10 |
| 3^rd^ primary antibody incubation | anti-c-Myc (Abcam Cat# ab32072, RRID: AB_731658; 1:50) | 30 |
| 3^rd^ secondary antibody incubation | anti-Rabbit HRP (Agilent Cat# K4003, RRID: AB_2630375) | 10 |
| 3^rd^ signal amplification and fluorophore deposition | TSA Opal 570 (PerkinElmer FP1487001KT; 1:100) | 10 |
| 4^th^ epitope retrieval (antibody stripping) | pH 9 HIER (low power) | 10 |
| 4^th^ epitope blocking | BSA | 10 |
| 4^th^ primary antibody incubation | anti-CD20 (Agilent Cat# M0755, RRID: AB_2282030; 1:2000) | 30 |
| 4^th^ secondary antibody incubation | anti-Mouse HRP | 10 |
| 4^th^ signal amplification and fluorophore deposition | TSA Opal 540 (PerkinElmer FP1487001KT; 1:100) | 10 |
| 5^th^ epitope retrieval (antibody stripping) | pH 9 HIER (high power) | 5 |
| 5^th^ epitope retrieval (continued) | pH 9 HIER (low power) | 10 |
| 5^th^ epitope blocking | BSA | 10 |
| 5^th^ primary antibody incubation | anti-Ki67 (Agilent Cat# M7240, RRID:AB_2142367; 1:50) | 45 |
| 5^th^ secondary antibody incubation | anti-Mouse HRP | 10 |
| 5^th^ signal amplification and fluorophore deposition | TSA Opal 620 (PerkinElmer FP1487001KT; 1:100) | 10 |
| Final antibody stripping | pH 9 HIER (low power) | 10 |
| Counterstaining | DAPI (PerkinElmer FP1490; 1:10 in antibody diluent Dako DKO.S302283) | 5 |
| Mounting | CC mount (Sigma C9368) | 10 |
|  |  |  |
| NUH – National University Hospital, CMMC - Chi-Mei Medical Center, TMA – tissue microarray, HRP – horseradish peroxidase, TSA – tyramide signal amplification, HIER – heat-induced epitope retrieval, BSA – bovine serum albumin, DAPI – 4′,6-diamidino-2-phenylindole. | | |

| Supplementary table 17. Automated multiplexed fluorescent immunohistochemistry (mfIHC) staining protocol performed on the SGH, MDA and BCA cohort TMA. | | |
| --- | --- | --- |
| Procedure | Reagent (product; dilution) | Duration (min.) |
| Bake at 60 °C |  | 20 |
| Dewax | Leica Biosystems Bond Dewax Solution (AR9222) | BondMax default step |
| 1^st^ epitope retrieval (BondMax) | Leica Biosystems Bond Epitope Retrieval Solution 2 (AR9640) | 20 |
| Peroxide Block | BOND Polymer Refine Detection Kit (DS9800) | 10 |
| 1^st^ Primary antibody incubation | anti-c-Myc (Abcam Cat# ab32072, RRID: AB_731658; 1:50) | 20 |
| 1^st^ Post Primary | BOND Polymer Refine Detection Kit (DS9800) | 8 |
| 1^st^ Polymer | BOND Polymer Refine Detection Kit (DS9800) | 8 |
| 1^st^ signal amplification and fluorophore deposition | TSA Opal 520 (PerkinElmer FP1487001KT; 1:100) | 10 |
| 2^nd^ epitope retrieval (BondMax) | Leica Biosystems Bond Epitope Retrieval Solution 1 (AR9961) | 15 |
| 2^nd^ primary antibody incubation | anti-CD20 (Agilent Cat# M0755, RRID: AB_2282030; 1:1000) | 20 |
| 2^nd^ Post Primary | BOND Polymer Refine Detection Kit (DS9800) | 8 |
| 2^nd^ Polymer | BOND Polymer Refine Detection Kit (DS9800) | 8 |
| 2^nd^ signal amplification and fluorophore deposition | TSA Opal 540 (PerkinElmer FP1494001KT; 1:100) | 10 |
| 3^rd^ epitope retrieval (BondMax) | Leica Biosystems Bond Epitope Retrieval Solution 1 (AR9961) | 20 |
| 3^rd^ primary antibody incubation | anti-Bcl-6 (Leica, LN22, NCL-564; 1:50) | 20 |
| 3^rd^ Post Primary | BOND Polymer Refine Detection Kit (DS9800) | 8 |
| 3^rd^ Polymer | BOND Polymer Refine Detection Kit (DS9800) | 8 |
| 3^rd^ signal amplification and fluorophore deposition | TSA Opal 620 (PerkinElmer FP1495001KT; 1:100) | 10 |
| 4^th^ epitope retrieval (BondMax) | Leica Biosystems Bond Epitope Retrieval Solution 2 (AR9640) | 20 |
| 4^th^ primary antibody incubation | anti-Bcl-2 (Agilent Cat# M0887, RRID: AB_2064429; 1:100) | 20 |
| 4^th^ Post Primary | BOND Polymer Refine Detection Kit (DS9800) | 8 |
| 4^th^ Polymer | BOND Polymer Refine Detection Kit (DS9800) | 8 |
| 4^th^ signal amplification and fluorophore deposition | TSA Opal 690 (PerkinElmer FP1497001KT; 1:100) | 10 |
| Counterstaining | DAPI (PerkinElmer FP1490; 1:10 in antibody diluent Dako DKO.S302283) | 5 |
| Mounting | ProLong Diamond Antifade Mountant, CC mount (Life Technologies) |  |
|  |  |  |
| SGH – Singapore General Hospital, MDA – MD Anderson Cancer Center, BCA - British Columbia Cancer Agency, TMA – tissue microarray, HRP – horseradish peroxidase, TSA – tyramide signal amplification, HIER – heat-induced epitope retrieval, BSA – bovine serum albumin, DAPI – 4′,6-diamidino-2-phenylindole. | | |

# References

1. Ennishi D, Jiang A, Boyle M, Collinge B, Grande BM, Ben-Neriah S*, et al.* Double-Hit Gene Expression Signature Defines a Distinct Subgroup of Germinal Center B-Cell-Like Diffuse Large B-Cell Lymphoma. J Clin Oncol **2019**;37(3):190-201 doi 10.1200/JCO.18.01583.

2. Ennishi D, Mottok A, Ben-Neriah S, Shulha HP, Farinha P, Chan FC*, et al.* Genetic profiling of MYC and BCL2 in diffuse large B-cell lymphoma determines cell-of-origin–specific clinical impact. Blood **2017**;129(20):2760-70 doi 10.1182/blood-2016-11-747022.

3. Gavagnin E, Owen JP, Yates CA. Pair correlation functions for identifying spatial correlation in discrete domains. Phys Rev E **2018**;97(6-1):062104 doi 10.1103/PhysRevE.97.062104.

4. Kaufmann J, Biscio CAN, Bankhead P, Zimmer S, Schmidberger H, Rubak E*, et al.* Using the R Package Spatstat to Assess Inhibitory Effects of Microregional Hypoxia on the Infiltration of Cancers of the Head and Neck Region by Cytotoxic T Lymphocytes. Cancers (Basel) **2021**;13(8) doi 10.3390/cancers13081924.

5. Baddeley A, Turner R. spatstat: An R Package for Analyzing Spatial Point Patterns. J Stat Softw **2005**;12(6):1 - 42 doi 10.18637/jss.v012.i06.
